# Seroprevalence and risk factors for *Brucella* species and *Coxiella burnetii* exposure in a cross-sectional serosurvey of occupationally exposed groups in peri-urban Lomé, Togo

**DOI:** 10.1371/journal.pntd.0012657

**Published:** 2026-01-20

**Authors:** Charlotte L. Kerr, Akouda Patassi, Pidemnéwé S. Pato, Javier Guitian, Sylvie Audrey Diop, Imadidden Musallam, Punam Mangtani, Patrick Nguipdop-Djomo

**Affiliations:** 1 Department of Infectious Disease Epidemiology and International Health, London School of Hygiene and Tropical Medicine, London, United Kingdom; 2 Service des Maladies Infectieuses, Centre Hospitalier Universitaire Sylvanus Olympio, Lomé, Togo; 3 Laboratoire Central Vétérinaire de Lomé, Direction de l’Elevage, Lomé, Togo; 4 Veterinary Epidemiology, Economics and Public Health Group, WOAH Collaborating Centre in Risk Analysis and Modelling, Department of Pathobiology and Population Sciences, The Royal Veterinary College, Hatfield, United Kingdom; 5 UFR of Health Sciences, University of Thiès, Thiès, Senegal; UDLA: Universidad de Las Americas, ECUADOR

## Abstract

**Background:**

*Brucella* species and *Coxiella burnetii* have been detected in livestock populations in Togo. Populations exposed to livestock ruminants through their occupation may be at increased risk of infection.

**Methods/Principal findings:**

A cross-sectional serosurvey was conducted in 108 abattoir and 81 dairy farm workers (from 52 dairy farms) in peri-urban Lomé, Togo in 2019–2020. Structured questionnaires were used to collect data on participant livestock contact and dairy product consumption. Sera were tested using the Rose Bengal plate agglutination test (RBT) and the indirect *Brucella* IgG Enzyme-Linked Immunosorbent Assay (ELISA) for *Brucella*, and the IgG ELISA for *Coxiella burnetii* in Phase 1 and in Phase 2. Fresh bulk milk samples from farms were tested using an indirect milk ELISA for *Brucella* IgG. The association between seropositivity and exposure variables was examined using logistic regression with robust standard errors to account for site-level clustering. Eighteen workers (9.5%, 95% CI 5.5-16.0) were *Brucella* seropositive. Twenty-eight percent (95% CI 22.5-34.3) of workers were seropositive for *C. burnetii*. Twenty of fifty-one farms which gave milk samples tested positive for *Brucella* antibodies (39.2%, 95% CI 26.6 - 53.4%). Farmworkers had nearly twice the crude odds of being *Brucella* seropositive compared to abattoir workers (OR 1.93, 95% CI: 0.94-3.93, p = 0.07). After adjusting for age, working on farms with animal ill health, a positive milk test, participating in small ruminant husbandry and assisting with cattle abortion were all associated with increased odds of seropositivity. Farm and abattoir workers who consumed raw milk at least every month were more likely to be seropositive for Brucella species (OR 3.79, 95% CI: 2.34-6.13, p < 0.001) while participants who consumed fermented milk and cheese had greater odds of being seropositive for *C. burnetii* (OR 1.59, 95% CI: 1.26-2.00, p < 0.001 and OR 1.70, 95% CI: 0.97-2.98, p = 0.07 respectively).

**Conclusions:**

Livestock workers in peri-urban Lomé have been exposed to both *Brucella* and *Coxiella burnetii* bacteria. The widespread consumption of raw dairy products and lack of personal protective equipment (PPE) use is of concern as both dairy consumption and participation in animal husbandry activities have been seen to increase odds of seropositivity for both pathogens. A One Health prioritization of zoonotic disease would help to bring together the relevant sectors to adequately resource prevention and control of zoonoses of public health concern in Togo, which may particularly impact workers in close contact with animals.

## Author summary

Human and animal health are inextricably linked, particularly for those who live and work closely with animals. Brucellosis and Q fever are two zoonotic infections transmitted through animal contact and dairy product consumption, which cause non-specific fevers and for which diagnostic tests are lacking in many Low and Middle-Income Countries (LMIC) contexts. Previous studies have shown that both bacteria circulate in livestock in Togo. We undertook a survey in dairy farm and abattoir workers in peri-urban Lomé, Togo, and found that 9.5% and 28% of workers were seropositive for *Brucella* and *C. burnetii* respectively. We found that risk factors included animal husbandry practices and consumption of dairy products. Mitigating practices such as the use of PPE and boiling milk can protect livestock workers from these and other zoonotic diseases. A systematic One Health prioritization of zoonoses for Togo would enable a multisectoral approach and adequate resource allocation for prevention and control of these diseases.

## Introduction

Zoonotic pathogens maintained by ruminant reservoirs, such as *Brucella* species and *Coxiella burnetii*, are of concern in agricultural communities, particularly in low-income countries (LICs) where animals and humans frequently interact, infection status of livestock holdings is often unknown and controls are lacking. Workers in the livestock industry, including slaughterhouse workers, farmers of ruminants, animal healthcare workers and veterinarians, are particularly vulnerable to such zoonoses which not only impact health and wellbeing but also livelihoods through worker’s reduced capacity to do labour and through livestock productivity losses [1].

Brucellosis is transmitted through consumption of dairy products, via contact with infected animals and their bodily fluids, and inhalation of contaminated aerosols [2]. The main causative organisms are *Brucella abortus* and *Brucella melitensis*, the main reservoirs of which are cattle, and small ruminants respectively [3]. While commonly presenting clinically as a non-specific febrile illness, infection may result in more serious, chronic sequelae such as osteoarticular disease including spondylitis and osteomyelitis, neurological disease and endocarditis [4–6]. A recent study looking at peri-urban dairy production in West and Central Africa found a *Brucella* herd prevalence of 62% in dairy herds from the surroundings of the capital city of Togo, Lomé [7]. *Brucella melitensis* was isolated from cattle hygroma samples in this region (personal communication).

*Coxiella burnetii* is most commonly transmitted through inhalation of aerosolized particles, though dairy product consumption and direct contact with bodily fluids of infected animals are also implicated [8,9]. The disease caused, Q fever, may also present as acute fever, with a proportion of infections progressing to hepatitis, pneumonia, endocarditis and chronic fatigue [8,10,11]. A livestock study, in Northern Togo, found an individual *C. burnetii* seroprevalence of 16.1% in cattle, 16.2% in sheep, and 8.8% in goats [12].

More than half of people in Togo work in agriculture, despite a rapidly urbanising population [13]. Local livestock production systems are largely informal and may magnify risk of exposure to these pathogens due to unrestricted grazing and transhumance, leading to a high-level of mixing between species and across large areas, and manual milking and slaughter of animals with limited hygiene measures [14].

Due to the non-specific clinical presentation, and lack of awareness and laboratory tests for these diseases, they are often misdiagnosed as other febrile illness such as malaria and typhoid fever [8,10]. There is a scarcity of good quality data on the level of human exposure to *Brucella* species and *C. burnetii* in Togo [10,15]. An increased focus on assessing the risk in these populations is required to control the burden of such preventable, and often poverty-related diseases.

To our knowledge, no previous studies to ascertain the prevalence of *Brucella* or *C. burnetii* seropositivity have been undertaken in people who work with dairy cattle in Southern Togo. In this study we aim to focus on known at-risk populations, given the high prevalence of brucellosis in large ruminants in the maritime region of Togo, to ascertain the prevalence of past infection with *Brucella*, and *C. burnetii*. We used a cross-sectional serosurvey in abattoir and farm workers in the peri-urban area of Lomé, Togo. We also assessed risk factors for seropositivity to these pathogens.

## Methods

### Ethics statement

This study was approved by the Ethics Committee for Health Research (Comité de Bioéthique pour la Recherche en Santé) of the Ministry of Health of Togo (ref. 008/2019/CBRS du 14 mars 2019). The research was also approved by the London School of Hygiene and Tropical Medicine’s Observational Ethics Committee. Informed written consent was obtained from all literate participants. If the participant could not read or write, a witness also signed the consent form.

### Study design and setting

The cross-sectional serosurvey of abattoir workers and livestock keepers was conducted between December 2019 and March 2020. It was embedded in a larger project on brucellosis in animals in West and Central Africa which had surveyed 100 randomly sampled dairy farms in peri-urban Lomé in 2017–2018 [7]. Farmworkers were recruited from these and other farms in the three Western prefectures of the Maritime region which supply fresh cow milk to Lomé. Abattoir staff were additionally recruited at the municipal abattoir in Lomé.

### Participant selection

All staff employed by the abattoir and veterinary staff were selected for study participation given their small number. Eighty four of the 167 (50.3%) independent butchers, who were registered at this abattoir and enumerated for this study, were selected by simple random sampling, with replacement when selected sampled workers were unavailable or refused.

Dairy farms from the larger project were preferentially enrolled. However due to herd movement, replacement farms for those who had left the area were selected by animal health workers using convenience sampling. Up to 3 individuals were randomly selected from a list of workers on each farm. An information leaflet describing the study was provided in French. A fieldworker explained in a local language, including Fulani and Ewe, where participants did not read French. Written consent was obtained prior to interview and blood collection. If the participant could not read or write, a witness also signed the consent form. This sampling strategy was expected to be able to detect an estimated *Brucella* seroprevalence of 10% with 5% precision [12,16,17].

### Human data collection and sample processing

In-person interviews were carried out in the participant’s language using a structured questionnaire with close-ended questions, on tablet computers using Open Data Kit (ODK) by trained interviewers. The questionnaire consisted of three sections, respectively on farm-level risk factors, livestock contact, and consumption of livestock products (S1 Appendix).

The phlebotomist collected 4mL of peripheral venous blood. Samples were labelled with unique identification numbers linked to questionnaire data, transported to the laboratory (Institut National d’Hygiene, Lomé) in a cool box at 4–8°C and centrifuged to obtain serum on the same day.

All sera were tested for anti-*Brucella* antibodies using the Rose Bengal plate agglutination test (RBT) (APHA, UK) and then stored in freezers at −20°C. This assay detects both agglutinating and non-agglutinating IgM, IgG and IgA antibodies [18]. All samples were also tested after sample collection was complete using the indirect *Brucella* IgG Enzyme-Linked Immunosorbent Assay (ELISA) (Serion ELISA classic, Germany) as per manufacturer’s instructions.

Sera were also analysed for detection of IgG antibodies against *Coxiella burnetii* in either Phase 1, which predominates in chronic infection, or Phase 2, which predominates in acute infection, using the Serion ELISA classic (Institut Virion/Serion GmbH, Germany) and classified as positive, borderline or negative according to manufacturer’s instructions.

### Cow’s milk sample collection and processing

Samples of fresh bulk milk were collected in 15mL Falcon tubes from each farm visited, transported in a cool box (4-8°C) to the laboratory (Department de l’Elevage, Lomé) where they were aliquoted into 1.5mL cryotubes and stored in freezers at −20°C. All samples were analysed at the same time once sample collection was complete using the BRUCELISA-M kit, an indirect milk ELISA for *Brucella* IgG (APHA Scientific, UK).

### Statistical analysis

Data were analysed using Stata 17 (Statcorp, College Station, TX, USA). Cross-tabulations were used to describe participants. The outcomes were positive *Brucella* or *C. burnetii* serology, defined as any individual who tested positive to either RBT or *Brucella* IgG ELISA for *Brucella*, and either Phase 1 or Phase 2 ELISA for *C. burnetii.* Borderline results were considered seronegative. Prevalence was calculated for both *Brucella* and *C. burnetii* seropositivity and 95% confidence intervals were computed using cluster-robust standard errors, with clustering at the site (individual farm/abattoir) level, and chi-square tests were used to compare clinical symptoms by serological status.

**Fig 1:**
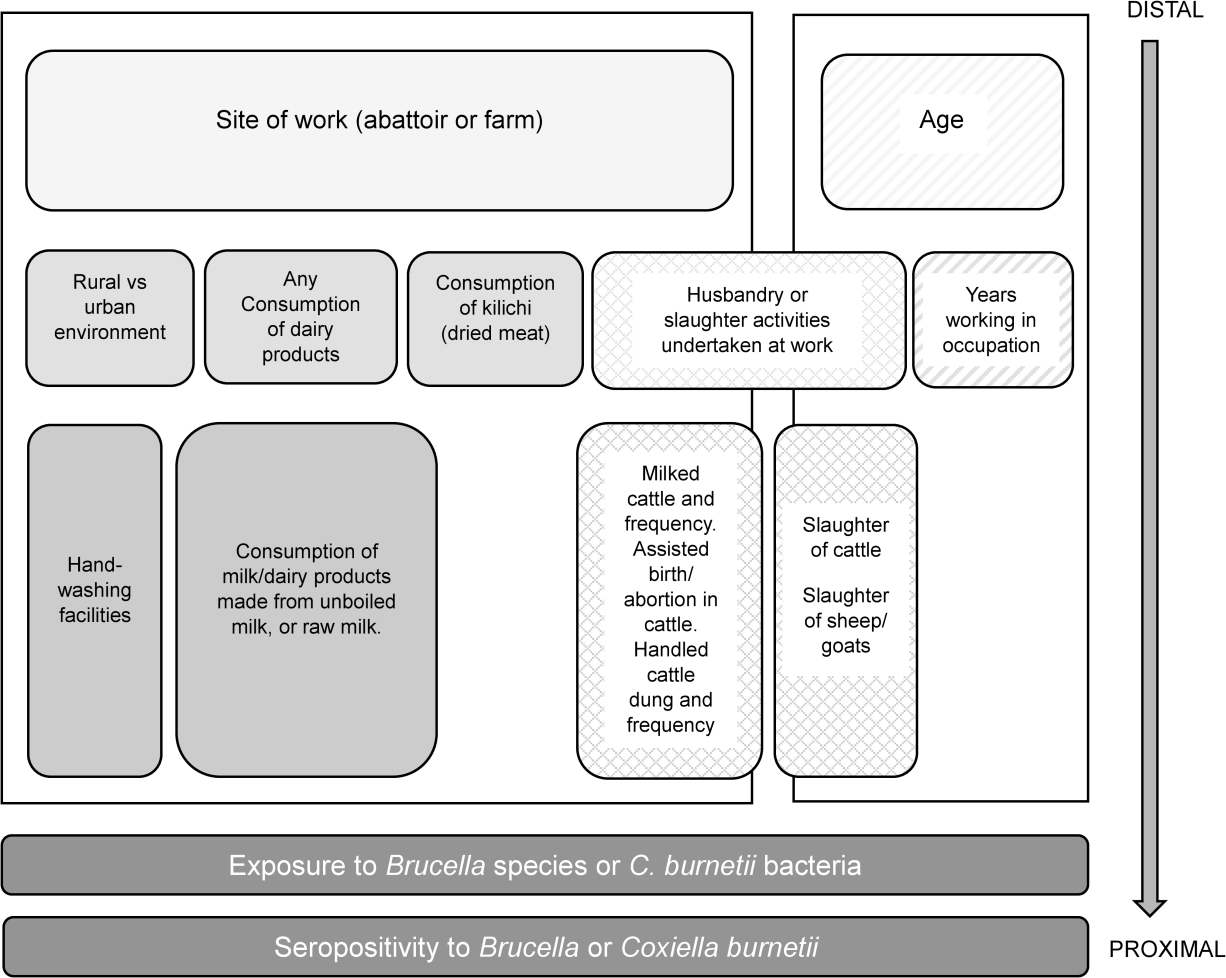
Hierarchical conceptual framework of the risk factors for seropositivity for *Brucella* and *Coxiella burnettii.* Legend: Shading becomes darker the more proximal a variable is to the outcome. Solid shading: variables on the pathway from site of work (abattoir or farm). Diagonal lines: variables on the pathway from age. Diamonds: variables on both pathways (site of work, and age).

A hierarchical conceptual framework (Fig 1) was developed to guide the analysis, grouping potential risk factors from distal to proximal. Age and site of work (abattoir or farm) were considered *a priori* confounders. The association between seropositivity and farm-related exposure variables in farmworkers, slaughter-related exposure variables in abattoir workers, and animal products consumption in all participants, were respectively investigated using logistic regression, with robust standard errors to account for site-level clustering. The likelihood ratio test was used to check for interaction between site of work (abattoir or farm) and the exposure variables. Due to data sparsity, multivariable analysis only examined the association between consumption variables and *Coxiella* seropositivity. The Wald test was used to assess evidence of association between the exposure variables and the outcome in the multivariable model. Models were assessed for multicollinearity and if detected the collinear variable which was least associated with the outcome was removed. Confounding was assessed by a 20% or more change in main exposure odds ratios when potential confounders were included in the model. In this paper, a p-value between 0.1 and 0.05 is interpreted as weak evidence, 0.001 < p < 0.05 is interpreted as good evidence and p < 0.001 as strong evidence.

## Results

### Participant characteristics including animal contact and dairy product consumption

Overall, 189 participants were recruited including 108 abattoir workers, and 81 farmworkers from 52 dairy farms. Three abattoir workers and one dairy farm refused to take part. Farm workers tended to be younger than the abattoir workers with less formal education (S1 Table).

Both abattoir and farm workers had commonly been involved in cattle and small ruminant slaughter ever in the past (51.0% and 56.4% with cattle, and 32.7 and 30.8% with small ruminants respectively) (S2 Table). Over a third (41.0%) of abattoir workers took part in animal husbandry in the last year. Use of PPE was rare, particularly in farmworkers (S2 Table).

Consumption of milk and dairy products was common including from raw milk (monthly consumption of 57% and 30% raw milk and dairy products respectively) (S3 Table).

### Dairy farm characteristics

There were high-levels of livestock ill health on farms, including ruminant abortions and cattle hygromas (S4 Table). Fifty-one (98.1%) farms gave bulk milk samples, of which 20 (39.2%, 95% CI 26.6 - 53.4%) tested positive for *Brucella antibodies*.

### Seroprevalence against *Coxiella burnetii* and *Brucella* species (S5 Table)

Seven abattoir workers (6.5%, 95% CI: 5.1-8.2) and 11 farmworkers (13.6%, 95% CI: 6.9-24.9), were seropositive for *Brucella*. *Coxiella* seroprevalence was higher at 31.5% (95% CI 24.6-39.3) in abattoir workers and 23.5% (95% CI: 15.4-34.1) in farmworkers. Eight (4.2%) participants were positive for antibodies against both pathogens, including six farmworkers.

### Risk factors for *Brucella* seropositivity

In a logistic regression model adjusted for age only, no variable was associated with *Brucella* seropositivity in the abattoir group (Table 1).

**Table 1:** Age-adjusted association^a^ between animal-contact risk factors and *Brucella* and *Coxiella* seropositivity in abattoir workers.

	*Coxiella burnetii*			*Brucella*		
	Number seropositive (%)	OR (95% CI)	p value*	Number seropositive (%)	OR (95% CI)	p value*
ONAF activity in abattoir^bc^						
No	28/91 (30.8)			5/91 (5.5)		
Yes	6/16 (37.5)	1.22 (0.39-3.76)	0.73	2/16 (12.5)	2.31 (0.39-13.66)	0.36
Slaughter activity in abattoir - all species^b^						
No	32/97 (33.0)			7/97 (7.2)		
Yes	2/10 (20.0)	0.50 (0.10-2.51)	0.4	0/10 (0)	na	na
Any butchery/skinning in abattoir - all species^b^						
No	11/44 (25.0)			2/44 (4.6)		
Yes	23/63 (36.5)	1.81 (0.76-4.30)	0.18	5/63 (7.9)	1.87 (0.35-10.18)	0.47
Any butchery/skinning in abattoir - sheep/goats^b^						
No	16/60 (26.7)			5/60 (8.3)		
Yes	13/28(46.4)	2.30 (0.89-5.92)	0.09	1/28 (3.6)	0.40 (0.04-3.60)	0.41
Years working in abattoir^b^						
0–15 years	14/50 (28.0)			3/50 (6.0)		
16 + years	17/45 (37.8)	1.23 (0.39-3.82)	0.72	3/45 (6.7)	0.61 (0.07-5.10)	0.65
Any husbandry activities with cattle^b^						
Never	23/75 (30.7)			6/75 (8.0)		
At least once	8/25 (32.0)	1.14 (0.42-3.05)	0.8	1/25 (4.0)	0.50 (0.06-4.40)	0.53
Any husbandry activities with sheep/goats^b^						
Never	22/81 (27.2)			7/81 (8.6)		
At least once	9/19 (47.4)	2.47 (0.88-6.96)	0.09	0/19 (0)	na	na

*P-values calculated from Wald test.

^a^Model is adjusted for age only, using logistic regression.

^b^Missing values for ONAF (Office National Des Abattoirs Et Frigorofiques) activity in abattoir n = 1; Slaughter activity in abattoir - all species n = 1; Any butchery/skinning in abattoir - all species n = 1; Any butchery/skinning in abattoir - sheep/goats n = 20; Years working in abattoir n = 13; Any husbandry activities with cattle n = 8; Any husbandry activities with sheep/goats n = 8.

^c^ONAF employees are abattoir staff who conduct other abattoir activities, such as cleaning of the abattoir after slaughter activities have finished including hosing down floors and walls.

There was some evidence that animal ill health on farms was associated with *Brucella* seropositivity in farmworkers in a model adjusted for age only (abortion in goats OR 5.22, 95% CI: 1.01-26.99, p = 0.05; death of young cattle OR 6.42 95% CI: 0.72-57.40, p = 0.1; death of young goats OR 5.81 95%CI: 1.0-33.81, p = 0.05) (Table 2). The odds of human *Brucella* seropositivity were five times higher on farms with a positive milk test for antibodies against *Brucella* species than those with a negative test in the same model (OR 5.15, 95% CI: 1.21-21.97, p = 0.03).

**Table 2:** Age-adjusted association^a^ between farm-level and animal husbandry risk factors and *Brucella* and *Coxiella* seropositivity in farmworkers.

	Coxiella burnetii			*Brucella*		
	Number seropositive (%)	OR (95% CI)	p value*	Number seropositive (%)	OR (95% CI)	p value*
**Farm-level variables**						
Number lactating cows						
<=median^b^	10/53 (18.9)			5/53 (9.4)		
> median	9/27 (33.3)	2.14 (0.76-6.02)	0.15	6/27 (22.2)	2.77 (0.64-11.90)	0.17
Sheep or not						
Absent	14/49 (28.6)			5/49 (10.2)		
Present	3/27 (11.1)	0.27 (0.07-1.13)	0.07	6/27 (22.2)	2.61 (0.54-12.59)	0.23
Goat or not						
Absent	11/44 (25.0)			5/44 (11.4)		
Present	6/34 (17.7)	0.66 (0.22-1.96)	0.45	6/34 (17.7)	1.74 (0.40-7.53)	0.46
Herd mixes with other herds						
No	4/17 (23.5)			1/17 (5.9)		
Yes	15/64 (23.4)	0.91 (0.29-2.89)	0.87	10/64 (15.6)	2.79 (0.33-23.19)	0.34
Presence of abortions in cows^c^						
No	6/32 (18.8)			2/32 (6.3)		
Yes	13/49 (26.5)	1.60 (0.56-4.60)	0.38	9/49 (18.4)	2.86 (0.53-15.34)	0.22
Presence of abortions in sheep^c^						
No	16/64 (25.0)			7/64 (10.9)		
Yes	3/17 (17.7)	0.66 (0.19-2.29)	0.51	4/17 (23.5)	2.00 (0.48-8.39)	0.35
Presence of abortions in goats^c^						
No	15/68 (22.1)			6/68 (8.8)		
Yes	4/13 (30.8)	1.73 (0.46-6.57)	0.42	5/13 (38.5)	5.22 (1.01-26.99)	0.05
Deaths of young in herd						
No	9/46 (19.6)			2/46 (4.4)		
Yes	10/35 (28.6)	1.76 (0.59-5.21)	0.31	9/35 (25.7)	6.42 (0.72-57.40)	0.1
Deaths of young sheep						
No	17/70 (24.3)			8/70 (11.4)		
Yes	2/11 (18.2)	0.73 (0.21-2.59)	0.63	3/11 (27.3)	2.13 (0.45-10.02)	0.34
Deaths of young goats						
No	16/72 (22.2)			7/72 (9.7)		
Yes	3/9 (33.3)	1.97 (0.49-7.97)	0.34	4/9 (44.4)	5.81 (1.0-33.81)	0.05
Presence of hygroma in cattle^d^						
Absent	na			5/52 (9.6)		
Present	na			6/29 (20.7)	2.38 (0.55-10.28)	0.25
*Brucella* ELISA milk test						
Negative	na			3/46 (6.5)		
Positive	na			8/33 (24.2)	5.15 (1.21-21.97)	0.03
**Animal contact**						
Years working on farm						
<=median^e^	10/42 (23.8)			6/42 (14.3)		
> median	9/38 (23.7)	0.88 (0.29-2.67)	0.82	5/38 (13.2)	1.55 (0.32-7.44)	0.58
Frequency of milking cattle						
Less than daily	5/17 (29.4)			1/17 (5.9)		
Daily	14/63 (22.2)	0.65 (0.19-2.30)	0.51	10/63 (15.9)	2.32 (0.22-24.17)	0.48
Assisting with birthing of cattle						
Never	4/17 (23.5)			1/17 (5.9)		
At least once	15/63 (23.8)	0.87 (0.27-2.77)	0.82	10/63 (15.9)	4.05 (0.51-32.12)	0.19
Assisting with abortion of cattle						
Never	7/30 (23.3)			2/30 (6.7)		
At least once	12/50 (24.0)	0.96 (0.37-2.50)	0.94	9/50 (18.0)	3.66 (0.85-15.84)	0.08
Frequency of other activities with cattle including handling dung						
Less than daily	6/19 (31.6)			1/19 (5.3)		
Daily	13/61 (21.3)	0.56 (0.16-1.91)	0.35	10/61 (16.4)	2.53 (0.31-20.94)	0.39
Any husbandry activities with sheep/goats						
Never	13/49 (26.5)			4/49 (8.2)		
At least once	6/31 (19.4)	0.58 (0.17-1.95)	0.38	7/31 (22.6)	3.9 (0.81-19.13)	0.09
Any cattle slaughter activities						
Never	9/34 (26.5)			8/34 (23.5)		
At least once	10/44 (22.7)	0.71 (0.27-1.89)	0.5	3/44 (6.8)	0.26 (0.07-1.01)	0.051
Any sheep/goat slaughter activities						
Never	14/54 (25.9)			8/54 (14.8)		
At least once	5/24 (20.8)	0.71 (0.22-2.32)	0.57	3/24 (12.5)	0.90 (0.23-3.52)	0.89

*P-values calculated from Wald test.

^a^Model is adjusted for age only, using logistic regression with clustered robust standard errors where individual farm is the cluster. As age of a participant and years working in their occupation were strongly correlated it was considered that adjusting for age will also largely account for confounding by the number of years working in these occupations.

^b^The median number of lactating cows was 10.

^c^Abortions reported to have occurred in the last year by the farmworker.

^d^Hygroma reported by farmworkers, not diagnosed by an animal health worker.

^e^The median number of years working on farms was 12 years.

There was weak evidence that participating in small ruminant husbandry was associated with nearly four times higher odds of *Brucella* seropositivity (age adjusted OR 3.9 95% CI 0.81-19.13, p = 0.09), as was assisting with cattle abortions (age adjusted OR 3.66, 95%CI: 0.85-15.84, p = 0.08).

In the combined farm and abattoir worker analysis farmworkers had nearly twice the odds of being seropositive compared to abattoir workers (age adjusted OR 1.93, 95% CI: 0.94-3.93, p = 0.07). Consuming raw cow’s milk on a monthly basis or more was associated with higher odds of *Brucella* seropositivity (OR 3.79, 95% CI: 2.34-6.13, p < 0.001) (Table 3) adjusted for age and site of work.

**Table 3:** Association between socio-demographic and dairy consumption factors and seropositivity to *Brucella* species in all participants^a.^

		*Brucella*		
		Number positive (%)	OR (95% CI)	p value*
**Education level**	None/coranic	14/99 (14.1)		
	Primary or above	4/89 (4.5)	0.34 (0.19-0.61)	<0.001
**Site type**	Abattoir	7/108 (6.5)		
	Farm	11/81 (13.6)	1.93 (0.94-3.93)	0.07
**Consume fermented milk including Caille**	No	4/63 (6.4)		
	Yes	14/126 (11.1)	1.29 (0.58-2.90)	0.53
**Consume cheese**	No	3/41 (7.3)		
	Yes	15/148 (10.1)	1.27 (0.47-3.38)	0.64
**Consume yoghurt**	No	5/60 (8.3)		
	Yes	13/129 (10.1)	1.32 (0.52-3.36)	0.56
**Consume raw cows dairy products**	Less than monthly	12/133 (9.0)		
	At least monthly	6/56 (10.7)	0.91 (0.29-2.88)	0.88
**Consume raw cows milk**	Less than monthly	3/81 (3.7)		
	At least monthly	15/108 (13.9)	3.79 (2.34-6.13)	<0.001
**Frequency of consuming any cows milk**	Less than monthly	2/54 (3.7)		
	Monthly	16/135 (11.9)	2.63 (1.97-3.49)	<0.001
**Consume kilichi**	Less than monthly	12/98 (12.2)		
	At least monthly	6/90 (6.7)	0.57 (0.22-1.46)	0.24
**Consume any milk/dairy from sheep/goats**	No	16/144 (11.1)		
	Yes	2/45 (4.4)	0.48 (0.22-1.05)	0.07

* P-values calculated from Wald test.

^a^Model is adjusted for age and type of site of work (abattoir or farm), using logistic regression with clustered robust standard errors where site of work is the cluster. Type of site of work was not adjusted for in the variable Site type. No interaction was observed between the site type of participants and any of the risk factors examined.

### Risk factors for *Coxiella* seropositivity

Adjusting for age and considering farm-level clustering, there was no evidence of an association with any livestock husbandry or livestock health variable in farmworkers (Table 2).

After adjusting for age only, there was no evidence that abattoir activities were associated with seropositivity, other than weak evidence of an association with butchery/skinning of small ruminants (p = 0.09) or in participating in any husbandry with sheep/goats (OR 2.47, 95% CI 0.88-6.96, p = 0.09) (Table 1).

The odds of *Coxiella* seropositivity in those who consumed fermented milk (OR 1.59, 95% CI: 1.26-2.00, p < 0.001) and in those who consumed cheese (OR 1.70, 95% CI: 0.97-2.98, p = 0.07) were greater than in those who did not when looking at all participants in a multivariable model considering dairy product consumption (Table 4).

**Table 4:** Baseline^a^ and Adjusted^b^ Odds Ratios for associations between dairy consumption factors and seropositivity to *Coxiella burnetii* in all participants.

		Number positive (%)	Baseline OR (95% CI)	p value*	Adjusted OR (95% CI)	p-value*
**Consume fermented milk including Caille**	No	16/63 (25.4)			1	
	Yes	37/126 (29.4)	1.64 (1.22-2.19)	0.001	1.59 (1.26-2.00)	<0.001
**Consume cheese**	No	9/41 (22.0)			1	
	Yes	44/148 (29.7)	1.69 (0.98-2.92)	0.06	1.70 (0.97-2.98)	0.066
**Frequency of consuming any cows milk**	Less than monthly	17/54 (31.5)			1	
	Monthly	36/135 (26.7)	0.97 (0.74-1.28)	0.86	0.73 (0.54-0.98)	0.04
**Ever consumed sheep/goat’s milk or dairy products**	No	41/144 (28.5)			1	
	Yes	12/45 (26.7)	0.76 (0.52-1.11)	0.16	0.75 (0.53-1.06)	0.099
**Site type**	Abattoir	34/108 (31.5)			1	
	Farm	19/81 (23.5)	0.76 (0.45-1.29)	0.32	0.66 (0.37-1.19)	0.17
**Consume yoghurt**	No	15/60 (25.0)				
	Yes	38/129 (29.5)	1.30 (0.83-2.04)	0.25		
**Consume raw cows dairy products**	Less than monthly	38/133 (28.6)				
	At least monthly	15/56 (26.8)	1.09 (0.47-2.53)	0.83		
**Consume raw cows milk**	Less than monthly	24/81 (29.6)				
	At least monthly	29/108 (26.9)	1.12 (0.8-1.58)	0.5		
**Consume kilichi**	Less than monthly	26/98 (26.5)				
	At least monthly	26/90 (28.9)	1.03 (0.66-1.61)	0.89		

*P-values calculated from Wald test.

^a^Model is adjusted for age and type of site of work (abattoir or farm), using logistic regression with clustered robust standard errors where site of work is the cluster. No interaction was observed between the site type of participants and any of the risk factors examined.

^b^Logistic regression model includes variables Consumption of fermented milk including Caille, Consumption of cheese, Frequency of consuming any cow’s milk, Ever consumed sheep/goat’s milk or dairy products, and the *a priori* variables age and type of site and clustered robust standard errors where site of work is the cluster. No interaction between exposure variables and whether a worker’s site of work was an abattoir or farm was seen.

### Fever history and related behaviours in all workers

Fever in the last year was commonly reported in the livestock workers (149/188, 79.3%) (S6 Table). Participants reporting fever in the last month were three times more likely to be *Brucella* seropositive (crude OR 3.63, 95% CI 1.21-10.90, p = 0.014) and no fever-related variable was associated with *Coxiella* seropositivity. The majority of participants who had experienced fever in the last year received no testing (82/141) or only for malaria (59/141). Antimalarials (76/142) and antibiotics (55/142) were commonly taken as treatment. The proportion of participants who had taken antimalarial drugs during a fever in the past year was higher in those seropositive for *Brucella* (68.8%) than those seronegative (51.6%) (p = 0.044).

## Discussion

We found, through a cross-sectional study of livestock workers, evidence of *Brucella* and *Coxiella burnetii* exposure in people with occupational exposure in peri-urban Lomé, with overall human seroprevalences of 9.5% and 28% respectively. Seroprevalence varied with site type, with a higher seroprevalence to *Brucella* on farms (13.6% compared to 6.5% in abattoirs) and to *C. burnetii* in abattoir workers (31.5% compared to 23.5% on farms). Bulk milk samples from associated cattle herds found that 39% (95% CI 27–53%) of farms also had *Brucella*-positive milk samples, lower than a previous study in Lomé’s peri-urban herds in 2017(62%, 95%CI 55–69%) [7]. Seropositivity to either pathogen was associated with the consumption of dairy products (raw milk in the case of *Brucella* and fermented milk and cheese in the case of Q fever), and ruminant husbandry (small ruminant husbandry and assisting with cattle abortion in farmworkers for *Brucella*, and small ruminant husbandry in abattoir workers for Q fever). The odds of brucellosis seropositivity in farmworkers was also increased with morbidity/mortality of owned ruminants (death of young cattle and goats, and abortion in goats). Considering this, the widespread ill-health in livestock including death of young animals and abortions which are consistent with brucellosis/coxiellosis, consumption of raw milk and dairy products, and lack of PPE (personal protective equipment) usage is of concern. However, due to the small sample size, analyses, other than that examining the association between *C. burnetii* seropositivity and dairy consumption, were limited to minimally adjusted models and as such results should be interpreted with caution.

Incidence of both infections varies greatly between contexts, including in different parts of the same country, and over time [10,15]. For this reason, it is difficult to make assumptions based on previous studies to inform policy. Our findings on *Brucella* seroprevalence were in contrast to a 2013 study in Northern Togo which found a seroprevalence of 2.4% in Fulani villagers, a comparable population to the farm workers in this study who had a seroprevalence of 13.6% [12]. This may be due to a lower prevalence in animals (9.2% in village cattle, 7.3% in transhumant cattle and 0% in small ruminants) though these results are from individual serology rather than bulk milk sampling [12]. The higher seroprevalence to *Coxiella burnetii* we note is aligned with the same study which found more than ten times the prevalence against *Coxiella* compared to *Brucella* in both Fulani and non-Fulani groups [12]. Similar, though not as marked, findings were seen in studies in Ethiopia, and Kenya [16,17,19]. Despite a higher seroprevalence of *Coxiella burnetii* in many African contexts, brucellosis is often given greater attention by health agencies and research funders [20,21]. Q fever can have severe health sequelae, being responsible for up to 5% of endocarditis cases globally, and if this disparity in seroprevalence is reflected in clinical burden then it is important to reassess Q fever as a priority [22].

There was a *C.burnetii* seroprevalence of 31.5% in the abattoir workers, despite the lack of abattoir-specific activities being associated with seropositivity. This was in line with de Boni et al who found no specific abattoir activity was a risk factor and hypothesised that workers were exposed when the bacterium was aerosolized during slaughterhouse activities [23]. Aerosols are a major transmission mode for *Coxiella burnetii* and this abattoir with its lack of ventilation provides ideal conditions for exposure of employees regardless of role [11]. The finding that Dutch cull workers were at greater risk of seroconverting if they worked indoors rather than outdoors supports this [24].

For both diseases infected livestock are the main reservoir, shedding bacteria in birth and abortion materials, milk, faeces, and urine [6,11]. Close contact with ruminants has been shown to be associated with seropositivity to *Brucella* and *C. burnetii*, as was seen for *Brucella* in our study in farmworkers who participated in small ruminant husbandry and assisting with cattle abortions, and for *C. burnetii* in abattoir workers who participated in small ruminant husbandry [25,26].

Abortion, birth of weak offspring and death of neonatal animals are symptoms of ruminant brucellosis and coxiellosis [27,28]. We demonstrated associations between human *Brucella* seropositivity and livestock ill-health markers (abortion in goats, and the death of young cattle on farm). Literature on this is limited and largely confined to animal abortion [29]. Animal-health variables such as abortion, and mortality in neonatal animals, may be indicators of herd/flock infection, and could be monitored using syndromic surveillance to mitigate risk to workers and livelihood losses. The association between *Brucella* seropositivity and presence of abortion in goats, and with participating in small ruminant husbandry is of particular interest as we know *B. melitensis* is circulating in ruminants in this region (personal communication). This highlights the complexity of transmission amongst species and to humans, and the need for multi-species studies.

This population consumed a high-level of bovine dairy products, including those sourced from raw milk. We found both consuming fermented milk and cheese were associated with *Coxiella burnetii* seropositivity. A 2019 study of hard sheep’s cheese purchased in Spanish supermarkets demonstrated the presence of viable *C. burnetii* in cheese samples using experimental inoculation of mice and culture in Vero cells, showing dairy products may be viable transmission vehicles [30]. However, there was no evidence that consumption of any cow’s milk or milk/dairy products from sheep/goats were associated with *Coxiella burnetii* seropositivity. Previously dairy products were considered to rarely contribute to *C. burnetii* transmission but the evidence is mixed with some studies finding an association [8,12,31].

Regular raw cow’s milk consumption increased the odds of *Brucella* seropositivity. Multiple studies have found consumption of unpasteurised milk to be a risk factor [32–34]. Consumption of small ruminant dairy products has also been shown to be a risk factor for brucellosis [35,36]. However, consumption of small ruminant dairy products isn’t culturally common in Togo, despite large numbers of these species, and those who did consume these weren’t found to have increased odds of *Brucella* seropositivity.

As sampling frames for agricultural occupational groups in Togo do not pre-exist, without a comprehensive census it is a challenge to sample a representative group. There are also practical limitations in carrying out a questionnaire-based survey in occupational groups with limited time for participation. Dean et al regarded a lack of detailed information on exposures as a limitation in thoroughly assessing risk [12]. While our questionnaire allowed more nuanced examination of risk factors it also limited the sample size. For this reason we could not perform certain analyses such as adjusting for other exposures of interest, including other animal husbandry and dairy consumption variables, in multivariable analysis for *Brucella* seropositivity.

There is a paucity of studies looking at linked animal and human data. Here we were able to link human data on brucellosis to milk sourced from associated cattle to show an association between presence of a positive bulk milk sample and human seropositivity. Bulk milk sampling is relatively affordable and easy and could be utilised in regular herd surveillance. To expand on this study, future multidisciplinary studies which conduct direct multi-species sampling, including small ruminants, would aid in elucidating transmission dynamics where human, animal and environmental health are highly interconnected. In the case of *Brucella*, this should include species identification and pathogen characterisation of any isolated bacteria. Such One Health studies could be expanded upon by examining other relevant populations and settings, and the economics of infection impacts and control.

There were low levels of PPE use in both groups. Many studies, though not all, have found PPE to be beneficial in protecting against both infections [37,38]. PPE use should be recommended, particularly adequate masks (P2/N95) in abattoir areas where aerosols are produced [39].

This population is affected by both zoonoses, and there is a need for preventative measures. Implementation should involve at-risk workers, trusted community leaders, health practitioners and policymakers using culturally sensitive, pragmatic and economically feasible approaches. Our study highlights the need for increased clinician awareness about zoonoses, and for relevant history taking including occupation, which might raise clinical suspicion, particularly as malaria prevalence declines [40]. Seropositive participants commonly experienced febrile disease though no symptoms were found to be associated, and this non-specific clinical picture hinders diagnosis by clinicians. Many seropositive participants were only tested for malaria and treated with antimalarials and non-specific antibiotics, similar to other studies [4,41]. In order to improve diagnosis healthworkers require resources to test and treat non-malarial illnesses, both to improve clinical outcomes and to reduce inappropriate prescribing which contributes to selection for antimicrobial resistance. In parallel to this, sensitisation of at-risk populations to improve awareness of these infections and mitigation strategies that could be utilised should be undertaken.

Due to the complicated interactions at the human-animal-environment interface in many zoonoses more complex interventions involving interdisciplinary teams tend to be required [20]. Integrated control of multiple zoonotic diseases, such as Q fever and brucellosis, through interventions targeting both the animal and human populations could have a dual benefit through improving animal health and livelihoods, while simultaneously decreasing the risk of human disease, to alleviate the cycle of poverty. Such integrated approaches would be more cost-effective by optimising resource use, especially when working with marginalised communities who are isolated from public services [21]. The findings of our study provide evidence which can be used to tailor such public health interventions against both *Brucella* and Q fever in occupational groups working with livestock. A number of countries in the region, including Côte d’Ivoire and Ghana, have recently undertaken a One Health Zoonotic Disease Prioritization exercise using the tool developed by the U.S. Centers for Disease Control and Prevention which aims to not only help prioritize disease of greatest public health impact but strengthen multisectoral collaboration and focus resource allocation, and Togo would benefit from a similar process [42,43].

## Supporting information

S1 Appendix Description of variables and variable creation. (DOCX)

S1 Table Characteristics of the study population, both overall and by site of work. (DOCX)

S2 Table Livestock contact, including by site of work. (DOCX)

S3 Table Dairy product and kilichi consumption, including by site of work. (DOCX)

S4 Table Farm characteristics of the 52 participating dairy farms: a) farm composition by animal and group, b) husbandry and management practices, c) morbidity and mortality in animals. (DOCX)

S5 Table Serological results of each test for *Brucella* species and *Coxiella burnetii.* (DOCX)

S6 Table Health and health seeking behaviours in participants in the previous 12 months. (DOCX)

S1 Text Questionnaire. (DOCX)

S2 Text Abstract and author summary (French translation). (DOCX)

S1 Checklist STROBE statement—Checklist of items that should be included in reports of cross-sectional studies. The filled checklist is based on the STROBE Statement-Checklist of items that should be included in reports of observational studies, developed by the STROBE Initiative, https://www.strobe-statement.org/.
(DOCX)
