# Supplementary material for: Seroprevalence and risk factors for Brucella species and Coxiella burnetii exposure in a cross-sectional serosurvey of occupationally exposed groups in peri-urban Lomé, Togo
Source: PLoS Negl Trop Dis. 2026 Jan 20;20(1):e0012657. doi: 10.1371/journal.pntd.0012657 (PMC12858067; doi:10.1371/journal.pntd.0012657)
Supplement: S1 Appendix — (DOCX) [file pntd.0012657.s001.docx]

**S1 Appendix: Description of variables and variable creation**

1. Farm-level variables: these included number of animals on farm by species, morbidity and mortality in animals including abortion and hygroma, animal movements and mixing. Animal numbers were dichotomised into none or some, where ownership was rare, or using the median as cut-off.
2. Livestock contact variables: Questions about livestock contact examined both husbandry and slaughter activity, with cattle, small ruminants and pigs examined separately, as well as asking about frequency of activity and duration, and use of protective equipment. To deal with issues of scarcity for some variables a number of categories were merged. Assisting with cattle abortions was a rare occurrence and was classified as never or at least once, whereas manual milking of cattle was commonly carried out on a daily basis and frequency was therefore classified as daily or less than daily/never.
3. Consumption of livestock product variables: Consumption of liquid milk in any form, processed dairy products, and dried meat were noted, with products from cattle and from small ruminants assessed separately. Information on the frequency of consumption and whether products had been boiled or not was collected.
4. Potential confounders: Data were collected on potential confounders including age, sex, ethnicity, religion and education.
5. Recent health and healthcare-seeking behaviours: Information was also gathered on this, including recent episodes of pyrexia, muscle or joint pain and night sweats
