# Supplementary material for: Seroprevalence and risk factors for Brucella species and Coxiella burnetii exposure in a cross-sectional serosurvey of occupationally exposed groups in peri-urban Lomé, Togo
Source: PLoS Negl Trop Dis. 2026 Jan 20;20(1):e0012657. doi: 10.1371/journal.pntd.0012657 (PMC12858067; doi:10.1371/journal.pntd.0012657)
Supplement: S1 Table — (DOCX) [file pntd.0012657.s002.docx]

**S1 Table: Characteristics of the study population, both overall and by site of work**

|  |  |  |  |  |  |
| --- | --- | --- | --- | --- | --- |
|  |  | **Total (%) (N=189)** | **Abattoir workers-n(%) (N=108)^b^** | **Farm workers-n(%) (N=81)** |  |
|  | **Site of work** |  |  |  |  |
|  | Abattoir | 108 (57.1) | na | na |  |
|  | Farm | 81 (42.9) | na | na |  |
|  | **Age (in years) ^ac^** |  |  |  |  |
|  | <25 | 33 (17.6) | 7 (6.5) | 26 (32.5) |  |
|  | 25-34 | 52 (27.7) | 23 (21.3) | 29 (36.3) |  |
|  | 35-44 | 45 (23.9) | 36 (33.3) | 9 (11.3) |  |
|  | 45+ | 58 (30.9) | 42 (38.9) | 16 (20.0) |  |
|  | **Sex** |  |  |  |  |
|  | Female | 10 (5.3) | 3 (2.8) | 7 (8.6) |  |
|  | Male | 179 (94.7) | 105 (97.2) | 74 (91.4) |  |
|  | **Education level ^a^** |  |  |  |  |
|  | None/Coranic | 99 (52.9) | 30 (27.8) | 69 (87.3) |  |
|  | Primary or above | 88 (47.1) | 78 (72.2) | 10 (12.7) |  |
|  | **Marital status ^a^** |  |  |  |  |
|  | Married/partnership | 146 (77.7) | 89 (83.2) | 57 (70.4) |  |
|  | Single | 42 (22.3) | 18 (16.8) | 24 (29.6) |  |
|  | **Religion ^a^** |  |  |  |  |
|  | Christian | 54 (28.7) | 52 (48.2) | 2 (2.5) |  |
|  | Muslim | 128 (68.1) | 53 (49.1) | 75 (93.8) |  |
|  | Other/unknown | 6 (3.2) | 3 (2.8) | 3 (3.8) |  |
|  | **Ethnicity** |  |  |  |  |
|  | Adja/Ewe/Mina/Kabye/Tem | 44 (23.3) | 40 (37.0) | 4 (4.9) |  |
|  | Hausa | 32 (16.9) | 31 (28.7) | 1 (1.2) |  |
|  | Fulani | 74 (39.2) | 0 (0.0) | 74 (91.4) |  |
|  | Other | 39 (20.6) | 37 (34.3) | 2 (2.5) |  |
|  | **Years working in occupation ^ac^** |  |  |  |  |
|  | 0-10 | 66 (37.5) | 30 (31.3) | 36 (45.0) |  |
|  | 11-20 | 57 (32.4) | 34 (35.4) | 23 (28.8) |  |
|  | 21-70 | 53 (30.1) | 32 (33.3) | 21 (26.3) |  |
|  | **Role in abattoir ^a^** |  |  |  |  |
|  | ONAF employees ^d^ | na | 14 (14.4) | na |  |
|  | Independent butcher | na | 62 (63.9) | na |  |
|  | Veterinary inspector | na | 10 (10.3 | na |  |
|  | Other | na | 11 (11.3) | na |  |
|  |  |  |  |  |  |

a Missing values for age n=1 (1 farmworker); education n=1 (1 farmworker); marital status n=1 (1 abattoir worker); religion n=1 (1 farmworker); years working in occupation n=13 (12 abattoir workers, 1 farmworker); Role in abattoir n=11

b One worker is an animal health worker who works both in abattoirs as well as visiting farms

c Age and years working in occupation Pearson correlation coefficient=0.74

d ONAF (Office National Des Abattoirs Et Frigorofiques) are abattoir staff who conduct other abattoir activities, such as cleaning of the abattoir after slaughter activities have finished including hosing down floors and walls

na: not applicable
