# Supplementary material for: Seroprevalence and risk factors for Brucella species and Coxiella burnetii exposure in a cross-sectional serosurvey of occupationally exposed groups in peri-urban Lomé, Togo
Source: PLoS Negl Trop Dis. 2026 Jan 20;20(1):e0012657. doi: 10.1371/journal.pntd.0012657 (PMC12858067; doi:10.1371/journal.pntd.0012657)
Supplement: S2 Table — (DOCX) [file pntd.0012657.s003.docx]

**S2 Table: Livestock contact, including by site of work**

| **ACTIVITY** | | Total (%) (N=189) | Abattoir workers n(%) (N=108) | Farm workers - n(%) (N=81) |
| --- | --- | --- | --- | --- |
| **ANIMAL CONTACT AT HOME** | |  |  |  |
| Animals in yard^a^ | | 120/185 (64.9) | 59/108 (54.6) | 61/77 (79.2) |
| Cattle in yard^a^ | | 69/185 (37.3) | 26/108 (24.1) | 43/77 (55.8) |
| Sheep in yard^a^ | | 69/184 (37.5) | 32/108 (29.6) | 37/76 (48.7) |
| Goats in yard^a^ | | 52/184 (28.3) | 28/108 (25.9) | 24/76 (31.6) |
| Pigs in yard^a^ | | 8/185 (4.3) | 6/108 (5.6) | 2/77 (2.6) |
| Dogs in yard^a^ | | 40/184 (21.7) | 13/108 (12.0) | 27/77 (35.1) |
| Animals sleep inside the home^a^ | | 46/185 (24.9) | 20/107 (18.7) | 26/78 (33.3) |
| Cattle sleep inside the home^a^ | | 7/183 (3.8) | 4/105 (3.8) | 3/78 (3.9) |
| Small ruminants sleep inside the home^a^ | | 11/183 (6.0) | 6/105 (5.7) | 5/78 (6.4) |
| Handled cattle carcasses in last year (died of unknown reasons) ^a^ | | 72/183 (39.3) | 28/105 (26.7) | 44/78 (56.4) |
| Handled sheep carcasses in last year (died of unknown reasons) ^a^ | | 47/183 (25.7) | 25/105 (23.8) | 22/78 (28.2) |
| Handled goat carcasses in last year (died of unknown reasons) ^a^ | | 38/183 (20.8) | 22/105 (21.0) | 16/78 (20.5) |
| **ANIMAL HUSBANDRY** | |  |  |  |
| Any animal husbandry ever^a^ | | 133/187 (71.1) | 53/107 (49.5) | 80/80 (100.0) |
| Any cattle husbandry ever^a^ | | 102/179 (57.0) | 26/101 (25.7) | 76/78 (97.4) |
| Any small ruminant husbandry ever^a^ | | 51/181 (28.2) | 20/101 (19.8) | 31/80 (38.8) |
| Last animal husbandry^a^ | none | 54/181 (29.8) | 54/101 (53.5) | 0/80 (0) |
|  | over 1 year ago | 10/181 (5.5) | 6/101 (5.9) | 4/80 (5.0) |
|  | in last year | 117/181 (64.6) | 41/101 (40.6) | 76/80 (95.0) |
| Ever milked cattle^a^ | | 79/181 (43.7) | 6/101 (5.9) | 73/80 (91.3) |
| Milk cattle daily^a^ | | 63/181 (34.8) | 0/101 (0) | 63/80 (78.8) |
| Birthed cattle^a^ | | 68/181 (37.6) | 5/101 (5.0) | 63/80 (78.8) |
| Birth cattle monthly^a^ | | 23/180 (12.8) | 3/101 (3.0) | 20/79 (25.3) |
| Assisted cattle abortion^a^ | | 54/181 (29.8) | 4/101 (4.0) | 50/80 (62.5) |
| Assist cattle abortion annually^a^ | | 32/181 (17.7) | 2/101 (2.0) | 30/80 (37.5) |
| Handle cattle dung^a^ | | 72/181 (39.8) | 20/101 (19.8) | 52/80 (65.0) |
| Handle cattle dung daily^a^ | | 48/181 (26.5) | 14/101 (13.9) | 34/80 (42.5) |
| Carry out other activities with cattle^a^ | | 82/179 (45.8) | 17/101 (16.8) | 65/78 (83.3) |
| Carry out other activities daily with cattle^a^ | | 66/178 (37.1) | 14/100 (14.0) | 52/78 (66.7) |
| Milked small ruminants^a^ | | 8/181 (4.4) | 3/101(3.0) | 5/80 (6.3) |
| Birthed small ruminants^a^ | | 22/181(12.2) | 9/101 (8.9) | 13/80 (16.3) |
| Assisted small ruminant abortion^a^ | | 18/181 (9.9) | 4/101 (4.0) | 14/80 (17.5) |
| Handled small ruminant dung^a^ | | 41/181 (22.7) | 18/101 (17.8) | 23/80 (28.8) |
| Other activities with small ruminants^a^ | | 36/181 (19.9) | 12/101 (11.9) | 24/80 (30.0) |
| Any pig husbandry ^a^ | | 8/181 (4.4) | 8/101 (7.9) | 0/80 (0) |
| Any use of personal protective equipment (PPE) during husbandry^ab^ | | 10/181 (5.5) | 7/101 (6.9) | 3/80 (3.8) |
| **ANIMAL SLAUGHTER** | |  |  |  |
| any animal slaughter^a^ | | 125/185 (67.6) | 79/107 (73.8) | 46/78 (59.0) |
| last animal slaughter^a^ | none | 60/176 (34.1) | 28/98 (28.6) | 32/78 (41.0) |
|  | over 1 year ago | 16/176 (9.1) | 5/98 (5.1) | 11/78 (14.1) |
|  | in the last year | 100/176 (56.8) | 65/98 (66.3) | 35/78 (44.9) |
| any cattle slaughter^a^ | | 94/176 (53.4) | 50/98 (51.0) | 44/78 (56.4) |
| daily cattle slaughter^a^ | | 44/176 (25.0) | 44/98 (44.9) | 0/78 (0) |
| any small ruminant slaughter^a^ | | 56/176 (31.8) | 32/98 (32.7) | 24/78 (30.8) |
| monthly small ruminaint slaughter^a^ | | 34/176 (19.3) | 26/98 (26.5) | 8/78 (10.3) |
| any pig slaughter^a^ | | 6/176 (3.4) | 5/98 (100.0) | 1/78 (1.3) |
| Any use of personal protective equipment (PPE) during slaughter^ac^ | | 5/176 (2.8) | 4/98 (4.1) | 1/78 (1.3) |

a Missing values for: animals in yard, cattle in yard, pigs in yard n=4 (same 4 farmworkers for all 3 variables); sheep in yard, goats in yard, dogs in yard n=5 (same 5 farmworkers for all 3 variables); Animals sleep inside the home n=4 (3 farmworkers, 1 abattoir workers); Cattle sleep inside the home, Small ruminants sleep inside the home n=6 (3 farmworkers, and 3 abattoir workers for both variables); Handled cattle carcasses in last year (died of unknown reasons); Handled sheep carcasses in last year (died of unknown reasons); Handled goat carcasses in last year (died of unknown reasons) n=6 (3 farmworkers, and 3 abattoir workers for all 3 variables); Any animal husbandry ever n=1 (1 farmworker, 1 abattoir worker); Any cattle husbandry ever n=10 (3 farmworkers and 7 abattoir workers); Any small ruminant husbandry ever, last animal husbandry, Ever milked cattle, milk cattle daily, birthed cattle, assisted cattle abortion, assist cattle abortion annually, handle cattle dung, handle cattle dung daily, Milked small ruminants, Birthed small ruminants, Assisted small ruminant abortion, Handled small ruminant dung, Other activities with small ruminants, Any pig husbandry n=8 (1 farmworker and 7 abattoir workers for all these variables); birth cattle monthly n=9 (2 farmworkers and 7 abattoir workers); Carry out other activities with cattle n=10 (3 farmworkers and 7 abattoir workers); Carry out other activities daily with cattle n=11 (3 farmworkers and 8 abattoir workers); Any use of personal protective equipment (PPE) during husbandry n=8 (1 farmworker and 7 abattoir workers); any animal slaughter n=4 (3 farmworkers and 1 abattoir workers); last animal slaughter, any cattle slaughter, daily cattle slaughter, any small ruminant slaughter, monthly small ruminant slaughter, any pig slaughter n=13 (3 farmworkers and 10 abattoir workers for all these variables); Any use of personal protective equipment (PPE) during slaughter n=13 (3 farmworkers and 10 abattoir workers for all these variables)

b Three participants protected their eyes during cattle husbandry (1 always, 2 rarely), 7 protected their hands during cattle husbandry (4 always, 1 often, 2 rarely), and 5 protected their mouth during cattle husbandry (2 often, 3 rarely). Two participants protected their eyes during small ruminant husbandry (2 rarely), 2 protected their hands during small ruminant husbandry (1 always, 1 often), and 2 protected their mouth during small ruminant husbandry (2 rarely).

c Two participants protected their eyes during slaughter (2 often), 1 protected their hands during slaughter (1 often), and 4 protected their mouth during slaughter (1 always, 2 often, 1 rarely).
