# Supplementary material for: Seroprevalence and risk factors for Brucella species and Coxiella burnetii exposure in a cross-sectional serosurvey of occupationally exposed groups in peri-urban Lomé, Togo
Source: PLoS Negl Trop Dis. 2026 Jan 20;20(1):e0012657. doi: 10.1371/journal.pntd.0012657 (PMC12858067; doi:10.1371/journal.pntd.0012657)
Supplement: S3 Table — (DOCX) [file pntd.0012657.s004.docx]

**S3 Table: Dairy product and kilichi consumption, including by site of work**

|  |  |  |  |  |  |  |  |
| --- | --- | --- | --- | --- | --- | --- | --- |
|  |  | | | Total (%) N=189 | Abattoir workers (%) N=108 | Farmworkers (%) N=81 |  |
|  | Consume dairy products | Caille/fermented milk | | 126/189 (66.7) | 53/108 (49.1) | 73/81 (90.1) |  |
|  |  | Milk | | 145/189 (76.7) | 68/108 (63.0) | 77/81 (95.1) |  |
|  |  | Yoghurt | | 129/189 (68.3) | 79/108 (73.2) | 50/81 (61.7) |  |
|  |  | Cheese | | 148/189 (78.3) | 81/108 (75.0) | 67/81 (82.7) |  |
|  |  | Ice-cream | | 58/189 (30.7) | 24/108 (22.2) | 34/81 (42.0) |  |
|  |  | Cream | | 56/189 (29.6) | 32/108 (29.6) | 24/81 (29.6) |  |
|  |  | Butter | | 76/189 (40.2) | 22/108 (20.4) | 54/81 (66.7) |  |
|  |  | Consume raw cow dairy products | Less than monthly or never | 133/189 (70.4) | 89/108 (82.4) | 44/81 (54.3) |  |
|  |  |  | monthly or more | 56/189 (29.6) | 19/108 (17.6) | 37 /81(45.7) |  |
|  |  | Consume boiled cow dairy products | Less than monthly or never | 105/189 (55.6) | 73/108 (67.6) | 32/81 (39.5) |  |
|  |  |  | monthly or more | 84/189 (44.4) | 35/108 (32.4) | 49/81 (60.5) |  |
|  |  | Consume cow dairy products of unknown boiling | Less than monthly or never | 145/189 (76.7) | 89/108 (82.4) | 56/81 (69.1) |  |
|  |  |  | monthly or more | 44/189 (23.3) | 19/108 (17.6) | 25/81 (30.9) |  |
|  | Consume milk | Drink raw cow milk | Never | 52/189 (27.5) | 46/108 (42.6) | 6/81 (7.4) |  |
|  |  |  | monthly or less | 56/189 (29.6) | 48/108 (44.4) | 8/81 (9.9) |  |
|  |  |  | Weekly or more | 81/189 (42.9) | 14/108 (13.0) | 67/81 (82.7) |  |
|  |  | Drink boiled cow milk^a^ | Never | 47/188 (25.0) | 26/107 (24.3) | 21/81 (25.9) |  |
|  |  |  | monthly or less | 80/188 (42.6) | 64/107 (59.8) | 16/81 (19.8) |  |
|  |  |  | Weekly or more | 61/188 (32.5) | 17/107 (15.9) | 44/81 (54.3) |  |
|  |  | Drink cow milk of unknown boiling | Never | 79/189 (41.8) | 39/108 (36.1) | 40/81 (49.4) |  |
|  |  |  | monthly or less | 87 /189 (46.0) | 60/108 (55.6) | 27/81 (33.3) |  |
|  |  |  | Weekly or more | 23/189 (12.2) | 9/108 (8.3) | 14/81 (17.3) |  |
|  |  | Drink raw milk shortly after collection (eg, during miking of cows) | never | 107/189 (56.6) | 73/108 (67.6) | 34/81 (42.0) |  |
|  |  |  | at least monthly | 37/189 (19.6) | 32/108 (29.6) | 5/81 (6.2) |  |
|  |  |  | at least weekly | 45/189 (23.8) | 3/108 (2.8) | 42/81 (51.9) |  |
|  | Ever drink shoat milk | | | 23/189 (12.2) | 19/108 (17.6) | 4/81 (4.9) |  |
|  | Ever consume shoat dairy products | | | 31/189 (16.4) | 26/108 (24.1) | 5/81 (6.2) |  |
|  | Buy dairy products from street/bike vendors | | | 28/189 (14.8) | 7/108 (6.5) | 21/81 (25.9) |  |
|  | Consume kilichi monthly ^a^ | | | 90/188 (47.9) | 55/107 (51.4) | 35/81 (43.2) |  |
|  |  |  |  |  |  |  |  |

a Missing values for: Drink boiled cow milk n=1 (1 abattoir worker); Consume kilichi monthly n=1 (1 abattoir worker)
