# Supplementary material for: Seroprevalence and risk factors for Brucella species and Coxiella burnetii exposure in a cross-sectional serosurvey of occupationally exposed groups in peri-urban Lomé, Togo
Source: PLoS Negl Trop Dis. 2026 Jan 20;20(1):e0012657. doi: 10.1371/journal.pntd.0012657 (PMC12858067; doi:10.1371/journal.pntd.0012657)
Supplement: S4 Table — (DOCX) [file pntd.0012657.s005.docx]

**S4:** **Table: Farm characteristics of the 52 participating dairy farms: a) farm composition by animal and group, b) husbandry and management practices, c) morbidity and mortality in animals.**

|  |  |  |  |  |  |  |
| --- | --- | --- | --- | --- | --- | --- |
|  |  | | **MEDIAN (RANGE)** | **NUMBER OF FARMS: n/N (%)** |  |  |
|  | **FARM COMPOSITION BY ANIMAL AND GROUP** | |  |  |  |  |
|  | Number lactating cows | | 10 (1-70) |  |  |  |
|  | Number dry cows | | 10 (0-100) |  |  |  |
|  | Number bulls | | 3 (1-26) |  |  |  |
|  | Herd size | | 42 (17-153) |  |  |  |
|  | Goats present on farm | |  | 21/51 (41.2)^a^ |  |  |
|  | Number of goats, if owned | | 10 (2-20) |  |  |  |
|  | Sheep present on farm | |  | 19/51 (37.3)^a^ |  |  |
|  | Number of sheep, if owned | | 8 (1-30) |  |  |  |
|  | **HUSBANDRY AND MANAGEMENT PRACTICES** | |  |  |  |  |
|  | Transhumance practiced | |  | 5/52 (9.6) |  |  |
|  | Mixing with other herds | Never |  | 11/52 (21.2) |  |  |
|  |  | Sometimes |  | 27/52 (51.9) |  |  |
|  |  | Regularly |  | 14/52 (26.9) |  |  |
|  | Herd ever mixes with goats and/or sheep | |  | 8/52 (15.4) |  |  |
|  | **MORBIDITY/MORTALITY IN ANIMALS** | |  |  |  |  |
|  | Abortion in cows (in last year) | | 1 (1-20) | 33/52 (63.5) |  |  |
|  |  | Abortion in sheep (in last year) | 0 (0-10) | 11/52 (21.2) |  |  |
|  |  | Abortion in goats (in last year) | 0 (0-7) | 11/52 (21.2) |  |  |
|  |  | Hygroma in cattle (at time of visit) | 0 (0-20) | 18/52 (34.6) |  |  |
|  |  | Deaths in herd (last 30 days) |  | 28/52 (53.9) |  |  |
|  |  | Deaths in young in herd (last 30 days) | 0 (0-7) | 24/52 (46.2) |  |  |
|  |  | Deaths in adults in herd (last 30 days) | 0 (0-4) | 19/52 (36.5) |  |  |
|  |  | Deaths in sheep (last 30 days) |  | 9/52 (17.3) |  |  |
|  |  | Deaths in young sheep (last 30 days) | 0 (0-6) | 7/52 (13.5) |  |  |
|  |  | Deaths in adult sheep (last 30 days) | 0 (0-10) | 8/52 (15.4) |  |  |
|  |  | Deaths in goats (last 30 days) |  | 10/52 (19.2) |  |  |
|  |  | Deaths in young goats (last 30 days) | 0 (0-8) | 8/52 (15.4) |  |  |
|  |  | Deaths in adult goats (last 30 days) | 0 (0-20) | 9/52 (17.3) |  |  |
|  |  | Milk ELISA for *Brucella* (positive) |  | 20/51 (39.2)^b^ |  |  |
|  |  |  |  |  |  |  |

a: 1 farm did not answer this question

b: 1 farm declined to provide a milk sample
