# Supplementary material for: Seroprevalence and risk factors for Brucella species and Coxiella burnetii exposure in a cross-sectional serosurvey of occupationally exposed groups in peri-urban Lomé, Togo
Source: PLoS Negl Trop Dis. 2026 Jan 20;20(1):e0012657. doi: 10.1371/journal.pntd.0012657 (PMC12858067; doi:10.1371/journal.pntd.0012657)
Supplement: S5 Table — (DOCX) [file pntd.0012657.s006.docx]

**S5 Table: Serological results of each test for *Brucella* species and *Coxiella burnetii***

|  |  | Number of seropositives (%) N=189 |
| --- | --- | --- |
| *Brucella* serology | RBT | 4 (2.1) |
|  | IgG ELISA | 10 (5.3) |
|  | Both tests | 4 (2.1) |
|  | At least one test | 18 (9.5) |
| *Coxiella burnetii* serology | COX1 | 4 (2.1) |
|  | COX2 | 21 (11.1) |
|  | Both tests | 28 (14.8) |
|  | At least one test | 53 (28.0) |

* RBT= Rose Bengal test, IgG ELISA= *Brucella* Immunoglobulin G enzyme-linked immunosorbent assay , COX1 ELISA = *Coxiella burnetii* Phase 1 Immunoglobulin G enzyme-linked immunosorbent assay , Cox2 ELISA = *Coxiella burnetii* Phase 2 Immunoglobulin G enzyme-linked immunosorbent assay
