# Supplementary material for: Seroprevalence and risk factors for Brucella species and Coxiella burnetii exposure in a cross-sectional serosurvey of occupationally exposed groups in peri-urban Lomé, Togo
Source: PLoS Negl Trop Dis. 2026 Jan 20;20(1):e0012657. doi: 10.1371/journal.pntd.0012657 (PMC12858067; doi:10.1371/journal.pntd.0012657)
Supplement: S6 Table — (DOCX) [file pntd.0012657.s007.docx]

**S6 Table: Health and health seeking behaviours in participants in the previous 12 months**

|  |  |  |  |  |  |  |
| --- | --- | --- | --- | --- | --- | --- |
|  |  | | n/N (%) (N=189) | Coxiella seropositive n (%) (N=53) | Brucella seropositive n (%) (N=18) |  |
|  | Fever in the last yr^a^ | | 149/188 (79.3) | 41/53 (77.4) | 16/18 (88.9) |  |
|  | Number of fevers in past year^a^ | None | 39/178 (21.9) | 12/50 (24.0) | 2/18 (11.1) |  |
|  |  | 1 | 41/178 (23.0) | 13/50 (26.0) | 6/18 (33.3) |  |
|  |  | 2-4 | 73/178 (41.0) | 16/50 (32.0) | 4/18 (22.2) |  |
|  |  | 5 + | 25/178 (14.0) | 9/50 (18.0) | 6/18 (33.3) |  |
|  | Last fever^a^ | No fever | 39/181 (21.6) | 12/52 (23.1) | 2/18 (11.1) |  |
|  |  | More than a month ago | 61/181 (33.7) | 18/52 (34.6) | 3/18 (16.7) |  |
|  |  | In the last month | 81/181 (44.8) | 22/52 (42.3) | 13/18 (72.2) |  |
|  | Fever of 2 weeks or more^a^ | | 45/181 (24.9) | 15/52 (28.9) | 5/18 (27.8) |  |
|  | Health provider sought in those with fever in the last year^a^ (N=149) | Hospital | 95/142 (66.9) | 29/40 (72.5) | 10/16 (62.5) |  |
|  |  | Pharmacy | 21/142 (14.8) | 4/40 (10.0) | 0/16 (0.0) |  |
|  |  | Traditional healer | 12/142 (8.5) | 3/40 (7.5) | 0/16 (0.0) |  |
|  |  | Ambulatory seller | 19/142 (13.4) | 3/40 (7.5) | 4/16 (25.0) |  |
|  |  | No care | 11/142 (7.8) | 4/40 (10.0) | 1/16 (6.3) |  |
|  | Test for fever in those with fever in the last year^a^ (N=149) | No | 82/141 (58.2) | 24/39 (61.5) | 10/16(62.5) |  |
|  |  | Yes, for malaria | 59/141 (41.8) | 15/39 (38.5) | 6/16 (37.5) |  |
|  | Treatment in those with fever in the last year^a^ (N=149) | Antimalarial | 76/142 (53.5) | 21/40(52.5) | 11/16 (68.8) |  |
|  |  | Antibiotic | 55/142 (38.7) | 15/40 (37.5) | 6/16 (37.5) |  |
|  |  | Paracetamol | 37/142 (26.1) | 8/40 (20.0) | 4/16 (25.0) |  |
|  |  | Traditional meds | 21/142 (14.8) | 4/40 (10.0) | 2/16 (12.5) |  |
|  | Symptoms during fever in those with fever in the last year^a^ (N=149) | Sweats and chills | 98/142 (69.0) | 28/40 (70.0) | 12/16 (75.0) |  |
|  |  | Bodyaches | 60/142 (42.3) | 18/40 (45.0) | 8/16 (50.0) |  |
|  |  | Lower backpain | 101/142 (71.1) | 31/40 (77.5) | 13/16 (81.3) |  |
|  |  | Jointpain | 107/142 (75.4) | 30/40 (75.0) | 12/16 (75.0) |  |
|  |  | Cough of 2 weeks | 40/142 (28.2) | 14/40 (35.0) | 5/16 (31.3) |  |
|  |  | Weightloss | 70/142 (49.3) | 19/40 (47.5) | 10/16 (62.5) |  |
|  |  | Headache of 2 weeks | 55/142 (38.7) | 13/40 (32.5) | 6/16 (37.5) |  |
|  |  | Abdominal pain | 47/142 (33.1) | 13/40 (32.5) | 6/16 (37.5) |  |
|  |  | Appetitie loss | 55/142 (38.7) | 17/40 (42.5) | 7/16 (43.8) |  |
|  |  | Testicular pain | 22/141 (15.6) | 3/40 (7.5) | 3/16 (18.8) |  |
|  |  |  |  |  |  |  |

a Missing values for: fever in the last year n=1; number of fevers in the past year n=11 (including 3 Coxiella seropositive workers); last fever, Fever of 2 weeks or more n=8 (including 1 Coxiella seropositive worker); Health provider sought in those with fever in the last year, Treatment in those with fever in the last year, Symptoms during fever in those with fever in the last year n=7 (including 1 Coxiella seropositive worker) with the exception of testicular pain where n=8 (including 1 Coxiella seropositive worker); Test for fever in those with fever in the last year n=8 (including 2 Coxiella seropositive workers)
