# Supplementary material for: Seroprevalence and risk factors for Brucella species and Coxiella burnetii exposure in a cross-sectional serosurvey of occupationally exposed groups in peri-urban Lomé, Togo
Source: PLoS Negl Trop Dis. 2026 Jan 20;20(1):e0012657. doi: 10.1371/journal.pntd.0012657 (PMC12858067; doi:10.1371/journal.pntd.0012657)
Supplement: S1 Text — (DOCX) [file pntd.0012657.s008.docx]

| Interviewer code | Interviewer initials | Site number | Household number | ID number |
| --- | --- | --- | --- | --- |
|  |  |  |  |  |

**Brucellosis in Lomé, Togo – Occupational questionnaire**

**[Before beginning the interview, please introduce yourself and confirm that the individual has been informed about the study and they have agreed to take part in the study]**

Hello, my name is ______ . I am working for a University in London called LSHTM and the Ministry of Livestock in Togo, and we are conducting a survey to investigate certain diseases which can be transmitted to people from livestock including cattle, sheep and goats. We want to find out how many people are exposed through their work and which factors increase their likelihood of being exposed. The information you provide us with will help us to understand how to control the disease better. You were selected at random from the workers on this farm/abattoir to participate in the survey if you are in agreement. I would like to ask you some questions about yourself which are considered relevant to this study. The questions usually take about 15 minutes and all information you provide us with will be confidential. Information will only be shared with other members of the survey team. You do not have to participate in the survey but we hope you will agree to as the information you provide us with is valuable in studying this disease. If I ask you any question you don't want to answer, just let me know and I will go on to the next question or you can stop the interview at any time. In case you need more information about the survey, you may contact the person listed on this card.

Section 1: Individual consent

[Before beginning the interview, please confirm that the prospective participant has been informed about the study, and has provided written informed consent (including witness signature if required))

| Date __/__/____ (ddmmyyyy) | |
| --- | --- |
| 1.1 Information sheet read and explained to participant (or legal guardian if minor) | 1-Yes [ ] 2-No [ ] |
| 1.2 Written consent given by the participant (or legal guardian if minor or witnessed if participant is illiterate) | 1 – Yes [ ] 2 – No [ ] |

Section 2: Individual identifying information of the participant

| 2.1 First name of participant |  |
| --- | --- |
| 2.2 Surname of participant |  |
| 2.3 Site number | _ _ _ |
| 2.4 Farm number | _ _ _ |
| 2.5 Participant ID number | _ _ _ |
| 2.6 Participant’s Main Phone number |  |
| 2.7. Participant's Alternative Phone number |  |
| 2.8 GPS coordinates |  |

Section 3: Demographic, occupational and farm information

To start, I would like to ask you some questions about you.

| 3.1 Respondent’s sex (interviewer note) | 1-Male [ ] 2-Female [ ] |
| --- | --- |
| 3.2 In what year were you born? | _ _ _ _  Don’t know [ ] |
| 3.3 How old were you at your last birthday (years)? | _ _ years  Don’t know [ ] |
| 3.4 What is your marital status? (If other specify) | Married/partnership [ ] Separated/Divorced [ ] Single [ ] Widow/widower [ ] Prefer not to say [] [ ] Other ______________________ |
| 3.5 What tribe/ethnic group do you belong to? (If other specify) | Kabye/Tem [ ] Adja-Ewe/Mina [ ] Para-Gourma/Akan [ ] Akposso/Akebou [ ] Ana-Ife [ ] Fulani/Peul [ ] Prefer not to answer [ ]  Other [ ] _________________ |
| 3.6 What is your religion?(If other specify) | Traditional [ ] Christian [ ] Muslim [ ] Prefer not to answer [ ]  Other [ ]__________________ |
| 3.7 What is the highest level of formal education you have achieved so far? | None/koranic [ ] Primary [ ] First stage Secondary [ ] Second stage secondary/ Technical/Vocational [ ]  Tertiary (university) [ ] |
| 3.8 Do you work on a dairy farm? If no continue to 3.22 | Yes [ ] No [ ] |

| 3.9 What is your main role on the farm? | Specify: |
| --- | --- |
|  | Classify:  Owner of farm (dairy cattle) [ ]  Farm worker (dairy cattle) [ ]  Veterinarian/animal health worker [ ]  Sale of livestock [ ]  Sale of dairy products [ ]  Processing of dairy products [ ]  Other [ ] _____________________ |
| 3.10 How long have you been working in this role? | __yrs__mnths  Don’t know [ ] |
| 3.11 Do you have any other source of income? If yes, specify and classify | Yes [ ] ____  No [ ]  Classify:  Other agricultural work [ ]  Management/technical/government [ ]  Business/commerce [ ]  Services (eg driver) [ ]  Employee (eg. Clerk) [ ]  Services (eg.driver) [ ]  Domestic worker [ ]  Skilled Manual (eg. electrician,mechanic) [ ]  Unskilled manual (e.g. construction, factory worker) [ ]  Other [ ] (specify):________________ |

| 3.12 Do you have animals on the farm? (including cows, goats, sheep, dogs, chickens). And if yes how many of the following animals does the farm have? (if none record 000, if unknown record 888) | Yes [ ] No [ ] |
| --- | --- |
|  | Lactating cows/buffalos: ___  Dry cows/buffalos: ___  Bulls:___  Other bovines:___  Horses, donkeys or mules:__  Goats:__  Sheep:__  Pigs:__  Chicken, duck or other poultry: __  Dogs: ___ |
| 3.13 Does the farm practice transhumance? (ie. The livestock are moved to different grazing grounds in a seasonal cycle) | Yes [] No [ ] Don’t know [ ] |
| 3.14 How often do the cattle come into contact with cattle from other herds eg during vaccination, grazing, veterinary treatment? | Regularly [ ] Sometimes [ ] never [ ] |
| 3.15 Do your cattle share grazing or enclosures with any of the following species? (tick all that apply) | Sheep [ ] Goats [ ] Pigs [ ] Horses [ ]  Don’t know [ ] None [ ] |
| 3.16 Have any of the cow’s aborted in the last 1 year to your knowledge? (if yes, specify how many) | Yes [ ] ___  No [ ] Don’t know [ ] |
| 3.17 If you have any other livestock on the farm have any of them aborted in the last 1 year to your knowledge? (if yes, specify how many and species if other animal) | Sheep: Yes [ ] __No [ ] Don’t know [ ]  Don’t own [ ]  Goats: Yes [ ] __No [ ] Don’t know [ ]  Don’t own [ ]  Other animals: Yes [ ] __________________  No [ ] Don’t know [ ] Don’t own [ ] |
| 3.18 Do any of the cow’s have joint swellings at present? (if yes, specify how many) | Yes [ ] ___  No [ ] Don’t know [ ] |
| 3.19 Have any of the herd died in the last 30 days? (if yes, specify how many adults and young) | Yes [ ] ____adults, ___young  No [ ]  Don’t know [ ] |
| 3.20 If you have any other livestock on the farm have any of them died in the last 30 day? (if yes, specify how many adults and young, and species if other animals) | Sheep: Yes [ ] ____adults, ___young No [ ]  Don’t know [ ] Don’t own [ ]  Goats: Yes [ ] ____adults, ___young No [ ]  Don’t know [ ] Don’t own [ ]  Other animals: Yes [ ] __species ____adults, ___young  No [ ] Don’t know [ ] Don’t own [ ] |
| 3.21 If you work on a dairy farm where is the milk you produce sold to (you can select more than one) | Direct to customers [ ]  Direct to vendors (resellers) [ ]  To a milk processing unit [ ]  To Milk bars or other transformers [ ]  Other(specify) [ ]:____________ |
| 3.22 Do you work at an abattoir? If no skip to section 4 | Yes [ ] No [] |
| 3.23 What is your job at the abattoir? | Butcher [ ]  Butcher’s assistant [ ]  Inspector of the Direction de l'elevage [ ]  Employee of the ONAF [ ]  Other [ ] _____________ |
| 3.24a Which of the following activities do you partake in with cattle and how many animals do you process per week doing this activity? | Slaughter: Yes [ ] ___ No [ ]  Skinning: Yes [ ] ___ No [ ]  Butchering: Yes [ ] ___ No [ ]  Inspection: Yes [ ] ___ No [ ]  ONAF activities: Yes [ ] No [ ]  No activity with this species: Yes [ ] No [ ]  Other: specify___ |
| 3.24b Which of the following activities do you partake in with sheep/goats and how many animals do you process per week doing this activity? | Slaughter: Yes [ ] ___ No [ ]  Skinning: Yes [ ] ___ No [ ]  Butchering: Yes [ ] ___ No [ ]  Inspection: Yes [ ] ___ No [ ]  ONAF activities: Yes [ ] No [ ]  No activity with this species: Yes [ ] No [ ]  Other: specify___ |
| 3.24c Which of the following activities do you partake in with pigs and how many animals do you process per week doing this activity? | Slaughter: Yes [ ] ___ No [ ]  Skinning: Yes [ ] ___ No [ ]  Butchering: Yes [ ] ___ No [ ]  Inspection: Yes [ ] ___ No [ ]  ONAF activities: Yes [ ] No [ ]  No activity with this species: Yes [ ] No [ ]  Other: specify___ |
| 3.25 How long have you been working in this role? (If less than a year, put 1) | __yrs  Don’t know [ ] |
| 3.26 Do you have any other source of income? If yes, specify | Yes [ ] ____  No [ ]  Classify:  Other agricultural work [ ]  Management/technical/government [ ]  Business/commerce [ ]  Services (eg driver) [ ]  Employee (eg. Clerk) [ ]  Services (eg.driver) [ ]  Domestic worker [ ]  Skilled Manual (eg. electrician,mechanic) [ ]  Unskilled manual (e.g. construction, factory worker) [ ]  Other [ ] (specify):________________ |

Section 4: Contact with livestock

Now, I would like to discuss with you the contacts that you may have had or usually have with livestock.

| 4.1 Have you ever participated in any livestock husbandry not including work at an abattoir (for example milking, feeding, or helping parturition of cows, goats, sheep or pigs? (if no continue to section 5) (Interviewer note: emphasise to the participant that this includes any livestock work even if only with a single animal) | Yes [ ]  No [ ] |
| --- | --- |
| 4.2 If yes, when was the last time you participated in these activities? | Less than one year ago [ ]  More than one year ago [ ]  I don’t remember [ ] |
| 4.3 Have you ever milked or helped to milk a cow or buffalo? (if no/don’t know continue to 4.6) | Yes [ ]  No [ ]  Don’t know [ ] |
| 4.4 If yes, what age were you when you first milked/helped to milk a cow/buffalo? | __yrs |
| 4.5 On average how often have you milked/helped to milk a cow/buffalo in the past 1 year/12 months? | Most days [ ]  At least once per week [ ]  At least once per month [ ]  Less than once a month [ ]  Have not done it in past 1 year [ ] |
| 4.6 Have you ever assisted in the calving of a cow or buffalo?  If yes answer questions 4.7-4.8, if no/don’t know continue to 4.9 | Yes [ ] No [ ] Don’t know [ ] |
| 4.7 If yes, what age were you when you first assisted in the calving of a cow/buffalo? | __yrs |
| 4.8 If yes, how often have you assisted in calving a cow/buffalo in the past 1 year? | Most days [ ]  At least once per week [ ]  At least once per month [ ]  Less than once a month [ ]  Have not done it in past 1 year [ ] |
| 4.9 Have you ever assisted during the abortion of a cow/buffalo or handled the abortion materials? (if no/don’t know continue to 4.12) | Yes [ ] No [ ] Don’t know [ ] |
| 4.10 If yes, what age were you when you first assisted in the abortion of a cow/buffalo or handled the abortion materials? | __yrs |
| 4.11 How often have you assisted during the abortion of a cow/buffalo or handled the abortion materials in the past 1 year/12 months? | Most days [ ]  At least once per week [ ]  At least once per month [ ]  Less than once a month [ ]  Have not done it in past 1 year [ ] |
| 4.12 Have you ever handled the dung of cattle/buffaloes, including when cleaning animal pens, using as fertiliser or for construction? If no/don’t know continue to 4.15 | Yes [ ] No [ ] Don’t know [ ] |
| 4.13 How old were you when you first handled the dung of cattle/buffalos? | ___yrs |
| 4.14 How often have you handled the dung of cattle/buffaloes in the past 1 year/12 months? | Most days [ ]  At least once per week [ ]  At least once per month [ ]  Less than once a month [ ]  Have not done it in past 1 year [ ] |
| 4.15 Have you ever taken part in any other activities with cattle/buffalos, for example their feeding, cleaning of their enclosures or herding them? If yes, specify  If no/don’t know continue to 4.18 | Yes [ ] ___  No [ ]  Don’t know [ ] |
| 4.16 How old were you when you first took part in any other activities with cattle/buffalos? | __yrs |
| 4.17 How often have you taken part in other activities with a cow/buffalo in the past 1 year/12 months? | Most days [ ]  At least once per week [ ]  At least once per month [ ]  Less than once a month [ ]  Have not done it in past 1 year [ ] |
| 4.18 When working with cattle/buffaloes do you wear anything to protect your eyes? If yes, specify | Yes [ ] ___  No [ ] |
| 4.19 If yes how often do you protect your eyes? | Always [ ] Often [ ] Rarely [ ] |
| 4.20 Do you wear anything to protect your hands when working with cattle/buffaloes? If yes, specify | Yes [ ] ___  No [ ] |
| 4.21 If yes how often do you protect your hands? | Always [ ] Often [ ] Rarely [ ] |
| 4.22 Do you wear anything to protect your mouth and nose when working with cattle buffaloes? If yes, specify | Yes [ ] ___  No [ ] |
| 4.23 If yes how often do you protect your mouth and nose? | Always [ ] Often [ ] Rarely [ ] |
| 4.24 Have you ever milked or helped to milk a goat or sheep?(if no continue to 4.27) | Yes [ ] No [ ] Don’t remember [ ] |
| 4.25 How old were you when you first milked or helped to milk a goat or sheep? | __yrs |
| 4.26 If yes how often have you milked/helped to milk a goat/sheep in the past 1 year/12 months? | Most days [ ]  At least once per week [ ]  At least once per month [ ]  Less than once a month [ ]  Have not done it in past 1 year [ ] |
| 4.27 Have you ever assisted in the birthing of a goat/sheep? (if no/don’t remember continue to 4.30) | Yes [ ] No [ ] Don’t remember [ ] |
| 4.28 How old were you when you first assisted in the birthing of a goat/sheep? | __yrs |
| 4.29 How often have you assisted in birthing a goat/sheep in the past 1year/12 months? | Most days [ ]  At least once per week [ ]  At least once per month [ ]  Less than once a month [ ]  Have not done it in past 1 year [ ] |
| 4.30 Have you ever assisted during the abortion of a goat/sheep or handled the abortion materials? (if no/don’t remember continue to 4.33) | Yes [ ] No [ ] Don’t remember [ ] |
| 4.31 How old were you when you first assisted during the abortion of a goat/sheep or handled the abortion materials? | __yrs |
| 4.32 How often have you assisted during the abortion of a goat/sheep or handled the abortion materials in the past 1 year/12 months? | Most days [ ]  At least once per week [ ]  At least once per month [ ]  Less than once a month [ ]  Have not done it in past 1 year [ ] |
| 4.33 In the past 12 months have you handled the dung of sheep/goats, including when cleaning animal pens, using as fertiliser or for construction? If no/don’t know continue to 4.36 | Yes [ ] No [ ] Don’t remember [ ] |
| 4.34 How old were you when you first handled the dung of sheep/goats? | __yrs |
| 4.35 How often have you handled the dung of sheep/goats in the past 1 year/12 months? | Most days [ ]  At least once per week [ ]  At least once per month [ ]  Less than once a month [ ]  Have not done it in past 1 year [ ] |
| 4.36 Have you ever taken part in any other activities with goats/sheep, for example their feeding,cleaning of their enclosures or herding them? If yes, specify  If no continue to 4.39 | Yes [ ] ___  No [ ]  Don’t remember [ ] |
| 4.37 How old were you when you first took part in other activities with goats/sheep? | __yrs |
| 4.38 How often have you taken part in other activities with goats/sheep in the past 1 year/12 months? | Most days [ ]  At least once per week [ ]  At least once per month [ ]  Less than once a month [ ]  Have not done it in past 1 year [ ] |
| 4.39 When working with sheep/goats do you wear anything to protect your eyes? If yes, specify | Yes [ ] ___  No [ ] |
| 4.40 If yes how often do you protect your eyes? | Always [ ] Often [ ] Rarely [ ] |
| 4.41 Do you wear anything to protect your hands when working with sheep/goats? If yes, specify | Yes [ ] ___  No [ ] |
| 4.42 If yes how often do you protect your hands? | Always [ ] Often [ ] Rarely [ ] |
| 4.43 Do you wear anything to protect your mouth and nose when working with sheep/goats? If yes, specify | Yes [ ] ___  No [ ] |
| 4.44 If yes how often do you protect your mouth and nose? | Always [ ] Often [ ] Rarely [ ] |
| 4.45 Have you ever taken part in any husbandry activities with pigs, for example their feeding or cleaning of their enclosures? If yes, specify | Yes [ ] _________  No [ ]  Don’t remember [ ] |
| 4.46 How old were you when you first took part in husbandry activities with pigs? | ______yrs |
| 4.47 How often have you taken part in husbandry activities with pigs? | Most days [ ]  At least once per week [ ]  At least once per month [ ]  Less than once a month [ ]  Have not done it in past 1 year [ ] |
| 4.48 Have you ever participated in the slaughter/butchery/skinning of any livestock (cows, goats, sheep or pigs? (Interviewer note: emphasise to the participant that this includes any slaughter/butchery/skinning even if only with a single animal, including during family celebrations) | Yes [ ] No [ ] |
| 4.49 If yes, when was the last time you participated in these activities? | Less than one year ago [ ]  More than one year ago [ ]  I don’t remember [ ] |
| 4.50 Have you ever slaughtered, butchered or skinned a cow/buffalo or assisted in that? (if no/don’t know continue to 4.15) | Yes [ ] No [ ] Don’t know [ ] |
| 4.51 What age were you when you first slaughtered, butchered or skinned a cow/buffalo or assisted in that? | __yrs |
| 4.52 If yes how often have you slaughtered or skinned a cow/buffalo or assisted in that in the past 1 year / 12 months? | Most days [ ]  At least once per week [ ]  At least once per month [ ]  Less than once a month [ ]  Have not done it in past 1 year [ ] |
| 4.53 Have you ever slaughtered, butchered or skinned a goat/sheep or assisted in that? (if no/don’t remember continue to 4.39) | Yes [ ] No [ ] Don’t remember [ ] |
| 4.54 How old were you when you first slaughtered, butchered or skinned a goat/sheep or assisted in that? | __yrs |
| 4.55 If yes how often have you slaughtered,butchered or skinned a goat/sheep or assisted in that in the past 1 year / 12 months? | Most days [ ]  At least once per week [ ]  At least once per month [ ]  Less than once a month [ ]  Have not done it in past 1 year [ ] |
| 4.56 Have you ever slaughtered, butchered or skinned a pig or assisted in that? (if no continue to 4.59) | Yes [ ] No [ ] Don’t remember [ ] |
| 4.57 How old were you when you first slaughtered, butchered or skinned a pig or assisted in that? | __yrs |
| 4.58 If yes how often have you slaughtered,butchered or skinned a pig or assisted in that in the past 1 year/12 months? | Most days [ ]  At least once per week [ ]  At least once per month [ ]  Less than once a month [ ]  Have not done it in past 1 year [ ] |
| 4.59 When participating in the slaughter/butchery/skinning of livestock do you wear anything to protect your eyes? If yes, specify. If never slaughtered/butchered/skinned livestock continue to 4.65 | Yes [ ] __________  No [ ] |
| 4.60 If yes how often do you protect your eyes? (Always: it would be extremely rare for this not to happen, Often: at least 50% of the time, Seldom: Less than 50% of the time) | Always [ ] Often [ ] Rarely [ ] |
| 4.61 Do you wear anything to protect your hands when participating in the slaughter/butchery/skinning of livestock? If yes, specify | Yes [ ] __________  No [ ] |
| 4.62 If yes how often do you protect your hands? (Always: it would be extremely rare for this not to happen, Often: at least 50% of the time, Seldom: Less than 50% of the time) | Always [ ] Often [ ] Rarely [ ] |
| 4.63 Do you wear anything to protect your mouth and nose when participating in the slaughter/butchery/skinning of livestock? If yes, specify | Yes [ ] __________  No [ ] |
| 4.64 If yes how often do you protect your mouth and nose? (Always: it would be extremely rare for this not to happen, Often: at least 50% of the time, Seldom: Less than 50% of the time) | Always [ ] Often [ ] Rarely [ ] |
| 4.65 Is it possible to wash your hands at the farm/abattoir? | Yes [ ]  No [ ]  Don’t know [ ] |
| 4.66 If yes what type of facility is this? | Bucket of water [ ] Running water [] ] Other [ ] specify ___ |
| 4.67 Do you normally wash your hands after taking part in work with livestock? | Always [ ] Often [ ] Rarely [ ] Never [ ] |
| 4.68 Do you normally use soap when you wash your hands? | Always [ ] Often [ ] Rarely [ ] Never [ ] |
| 4.69 In the past 12 months, have any animals ever spent time in the courtyard of the home or in the compound? This includes cows, goats, sheep, horses, donkeys, mules, poultry, pigs and dogs and other animals (if yes which of the following species and if yes to other animals, specify the species) | Cattle: Yes [ ] No [ ] Don’t know [ ]  Goats: yes [] No [] Don’t know [ ]  Sheep: Yes [ ] No [ ] Don’t know [ ]  Horses, donkeys or mule: Yes [ ] No [ ] Don’t know [ ]  Pigs: Yes [ ] No [ ] Don’t know [ ]  Chicken, Duck or other poultry: Yes [ ] No [ ] Don’t know [ ]  Dogs: Yes [ ] No [ ] Don’t know [ ]  Other animal: yes [ ] No [] Don’t know [ ] Specify: ___ |
| 4.70 In the past 12 months, has there been an occasion when any animals have spent the night inside the home (eg. In the kitchen, the storage room or the living room)? This includes cows, goats, sheep, horses, donkeys, mules, poultry, pigs and dogs and other animals(if yes which of the following species, and if yes to to other animals, specify the species) | Cattle: Yes [ ] No [ ] Don’t know [ ]  Goats: yes [] No [] Don’t know [ ]  Sheep: Yes [ ] No [ ] Don’t know [ ]  Horses, donkeys or mule: Yes [ ] No [ ] Don’t know [ ]  Pigs: Yes [ ] No [ ] Don’t know [ ]  Chicken, Duck or other poultry: Yes [ ] No [ ] Don’t know [ ]  Dogs: Yes [ ] No [ ] Don’t know [ ]  Other animal: yes [ ] No [] Don’t know [ ] Specify: ___ |
| 4.71 In the past 12 months have you handled or been in contact with the carcass, which has died of unknown reasons, of any animals? This includes cows, goats, sheep, horses, donkeys, mules, poultry, pigs and dogs and other animals (if yes to other animals, specify species) | Cattle: Yes [ ] No [ ] Don’t know [ ]  Goats: yes [] No [] Don’t know [] ]  Sheep: Yes [ ] No [ ] Don’t know  Pigs: yes [ ] No [ ] Don’t know [ ]  Dogs: Yes [ ] No [] Don’t know [ ]  Donkeys: Yes [ ] No [ ] Don’t know [] ]  Other animal: yes [ ] No [] Don’t know [ ] Specify: ___ |

Section 5: Consumption of livestock products

Now, I’m going to ask you about some of your eating habits.

| 5.1 Which of these dairy products do you consume from animal milk (cow, goat or sheep)? (tick all that apply) | Milk []  Fermented milk [ ]  Caille [ ]  Yoghurt [ ]  Cream [ ]  Cheese/Wangachi [ ]  Ice cream [ ]  Butter [ ]  None [ ]  Other (specify): ­­­­­­­­­­__________ |
| --- | --- |
| The following questions 5.2 – 5.13 refer to fresh liquid milk rather than milk made from powder (from the cow, goat or sheep) |  |
| 5.2 Approximately how often do you drink cow/buffalo milk in any form (for example Raw, Caillé or Fermented) which has not been boiled**?** This also includes milk that you mix in porridge or other meals. | Nearly every day [ ]  At least once per week [ ]  At least once a month [ ]  Less than once a month [ ]  Never [ ] |
| 5.3 Approximately how often do you drink cow/buffalo milk in any form (for example Raw, Caillé or Fermented), which has been boiled**?**  This also includes milk that you mix in porridge or other meals. | Nearly every day [ ]  At least once per week [ ]  At least once a month [ ]  Less than once a month [ ]  Never [ ] |
| 5.4 Approximately how often do you drink cow/buffalo milk in any form (for example Raw, Caillé or Fermented), that you are unsure if it is boiled or not? This also includes milk that you mix in porridge or other meals. | Nearly every day [ ]  At least once per week [ ]  At least once a month [ ]  Less than once a month [ ]  Never [ ] |
| 5.5 Approximately how often do you consume other dairy products like yoghurt, cream, butter or cheese, that you know has been made from Boiled cow/buffalo milk ? | Nearly every day [ ]  At least once per week [ ]  At least once a month [ ]  Less than once a month [ ]  Never [ ] |
| 5.6 Approximately how often do you consume other dairy products like yoghurt, cream, butter or cheese, that you know has been made from Unboiled cow/buffalo milk ? | Nearly every day [ ]  At least once per week [ ]  At least once a month [ ]  Less than once a month [ ]  Never [ ] |
| 5.7 Approximately how often do you consume other dairy products like yoghurt, cream, butter or cheese, that you do not know whether it has been made from Boiled or unboiled cow/buffalo milk ? | Nearly every day [ ]  At least once per week [ ]  At least once a month [ ]  Less than once a month [ ]  Never [ ] |
| 5.8 Approximately how often do you drink sheep/goats milk in any form (for example Raw, Caillé or Fermented) which has not been boiled? This also includes milk that you mix in porridge or other meals. | Nearly every day [ ]  At least once per week [ ]  At least once a month [ ]  Less than once a month [ ]  Never [ ] |
| 5.9 Approximately how often do you drink sheep/goats milk in any form (for example Raw, Caillé or Fermented), which has been boiled? This also includes milk that you mix in porridge or other meals. | Nearly every day [ ]  At least once per week [ ]  At least once a month [ ]  Less than once a month [ ]  Never [ ] |
| 5.10 Approximately how often do you drink sheep/goats milk in any form (for example Raw, Caillé or Fermented), that you are unsure if it is boiled or not? This also includes milk that you mix in porridge or other meals. | Nearly every day [ ]  At least once per week [ ]  At least once a month [ ]  Less than once a month [ ]  Never [ ] |
| 5.11 Approximately how often do you consume other dairy products like yoghurt, cream, butter or cheese, that you know has been made from Boiled sheep/goat’s milk? | Nearly every day [ ]  At least once per week [ ]  At least once a month [ ]  Less than once a month [ ]  Never [ ] |
| 5.12 Approximately how often do you consume other dairy products like yoghurt, cream, butter or cheese, that you know has been made from Unboiled sheep/goat’s milk? | Nearly every day [ ]  At least once per week [ ]  At least once a month [ ]  Less than once a month [ ]  Never [ ] |
| 5.13 Approximately how often do you consume other dairy products like yoghurt, cream, butter or cheese, that you do not know whether it has been made from Boiled or unboiled sheep/goats milk ? | Nearly every day [ ]  At least once per week [ ]  At least once a month [ ]  Less than once a month [ ]  Never [ ] |
| 5.14 Sometimes, some people consume small quantities of raw milk when taking part in certain activities, for example during the milking of cows, or when they collect milk from different flocks or farms, or even while they prepare the milk for sale/transformation. Have you ever drunk raw milk under similar circumstances? | Yes [ ] No [ ] |
| If yes to q 5.14 answer 5.15, if not skip to 5.16: |  |
| 5.15 On average, how often have you done that in the past 24 months? | Nearly every day [ ]  At least once per week [ ]  At least once a month [ ]  Less than once a month [ ]  Never [ ] |
| 5.16 Have you purchased dairy products from outside the farm in the past year? If no skip to 5.21 | Yes [ ] No [ ] |
| 5.18 If yes, which of the following have you purchased from outside the farm? (tick all that apply) | Fresh milk from cows/buffalo [ ]  Fermented milk from cow/buffalo [ ]  Fresh milk from sheep/goat [ ]  Fermented milk from sheep/goat [ ]  Yoghurt [ ]  Cheese, butter or ice-cream [ ]  Other milk based foodstuff [ ] |
| 5.19 How often do you purchase dairy products not from the farm? | At least once a week [ ] At least once a month [ ] At least once in the last 6 months [ ] At least once in the last year [ ] Don’t know [ ] |
| 5.20 Where do you purchase dairy products from which are from outside your farm? (tick all that apply) | Other household [ ]  Shop [ ]  Street vendor [ ]  Bike/itinerant vendor [ ]  Other (specify) [ ] |
| 5.21 Approximately how often do you consume dried meat (eg kilichi)? | Nearly every day [ ]  At least once per week [ ]  At least once a month [ ]  Less than once a month [ ]  Never [ ] |
|  |  |

Section 6: Health and health seeking behaviour

Now I’d like to ask you about what you do when you are ill.

| 6.1 Have you suffered from a fever in the last 12 months? If no, continue to section 7 | Yes [ ] No [ ] Don’t know [ ] |
| --- | --- |
| 6.2 Have you suffered from a fever which lasted for 2 weeks or longer? If yes, specify for how long (the most recent long fever) in weeks | Yes [ ]_____ No [ ] Don’t know [ ] |
| 6.3 How many episodes of fever have you suffered from in the last 12 months? | Specify ___ |
| 6.4 When was the last episode of fever you experienced? | In the last week [ ]  In the last 2 weeks [ ]  In the last month [ ]  In the last 3 months [ ]  In the last 6 months [ ]  In the last year [ ]  Don’t know [ ] |
| 6.5 Did you have any of these additional symptoms during or after a fever? If any other symptoms please specify | Intermittent sweating or chills (day or night): Yes [ ] No [ ]  Body aches for 2 weeks or longer: Yes [ ] No [ ]  Lower back pain: Yes [ ] No [ ]  Joint pains: Yes [ ] No [ ]  Cough for 2 weeks or longer: Yes [ ] No [ ]  Weight loss: Yes [ ] No [ ]  Headaches for 2 weeks or longer: Yes [ ] No [ ]  Abdominal pain for 2 weeks or longer: Yes [ ] No [ ]  Lack of appetite for 2 weeks or longer: Yes [ ] No [ ]  ONLY IF MALE, testicular pain: Yes [ ] No [ ]  Other: ____ |
| 6.6 If you experienced fever in the last year who did you visit to receive care? (tick all that apply) | Hospital/health centre [ ]  Drug shop/pharmacy [ ]  Traditional healer [ ]  Ambulatory sellers or automedication [ ]  Other [ ] specify ___  Did not seek help [ ] |
| 6.7 Did you have any tests done when you were suffering from a fever? If not a malaria test but known test please specify | Yes, for malaria [ ]  Yes, other [ ] Specify __  No [ ] |
| 6.9 Did you receive any medication when you experienced fever? | Antimalarials [ ]  Antibiotics [ ]  Ibuprofen [ ]  Paracetamol [ ]  Traditional medications [ ]  Other (specify):  No medications [ ]  Don’t know which [ ] |

Thank you very much for taking the time to reply to our questions. Now, the nurse would like to take a small blood sample. This sample will be tested in the laboratory to see if you may have been exposed in the past to some microbes that may be transmitted from livestock to humans that cause this disease, for example during contacts with them. The blood sample will only be tested for exposure to these microbes, and will not be tested for any other disease. After we have done the test, we will contact you to give you the result and to explain what it means and what you can do to better protect yourself.

Section 7: Consent for blood sample

| 7.1 Written consent for blood sample given by participant | Yes [ ] No [ ] |
| --- | --- |
| 7.2 Date and Time Blood sample collection | ddmmyyyy HH:MM |
| 7.3 Full participant ID |  |

Thank you one more time. We will contact you with the results after the blood sample has been examined in the laboratory. Meanwhile, if you have questions or concerns about this interview that you would like to discuss, you can contact us by sending a text message to the phone number or an electronic message to the email address mentioned in the information notice.
