# Supplementary material for: Seroprevalence and risk factors for Brucella species and Coxiella burnetii exposure in a cross-sectional serosurvey of occupationally exposed groups in peri-urban Lomé, Togo
Source: PLoS Negl Trop Dis. 2026 Jan 20;20(1):e0012657. doi: 10.1371/journal.pntd.0012657 (PMC12858067; doi:10.1371/journal.pntd.0012657)
Supplement: S2 Text — (DOCX) [file pntd.0012657.s009.docx]

**Résumé**

Introduction:

Des espèces de Brucella spp. et Coxiella burnetii ont été précédemment détectées dans le bétail au Togo. Les populations exposées aux ruminants d’élevage dans le cadre de leur profession peuvent être exposées à un risque accru d’infection.

Méthodes/Résultats principaux:

Une enquête sérologique transversale a été menée auprès de 108 travailleurs d'abattoirs et de 81 travailleurs de fermes laitières (provenant de 52 fermes laitières) de la zone périurbaine de Lomé, au Togo, en 2019-2020. Des questionnaires structurés ont été utilisés pour recueillir des données sur les contacts des participants avec le bétail et sur la consommation de produits laitiers. Les sérums ont été testés à l'aide du test d'agglutination sur plaque de rose Bengale (RBT) et du test immuno-enzymatique indirect Brucella IgG (ELISA) pour Brucella, et de l'ELISA IgG pour Coxiella burnetii en phase 1 et en phase 2. Le lait frais en vrac provenant des fermes a été testé à l'aide d'ELISA indirect pour les IgG du lait de Brucella. L’association entre la séropositivité et les variables d’exposition a été examinée à l’aide d’une régression logistique avec des erreurs standard robustes pour tenir compte du regroupement au niveau du site.

Dix-huit travailleurs (9,5 %, IC à 95 % 5,5-16,0) étaient séropositifs à Brucella. Vingt-huit pour cent (IC à 95 % 22,5-34,3) des travailleurs étaient séropositifs pour C. burnetii. Vingt des cinquante et une fermes laitières qui ont fourni des échantillons de lait ont été testées positives à Brucella (39,2 %, IC à 95 % 26,6 - 53,4 %).

Dans l'analyse univariée, les travailleurs de fermes laitières avaient près de deux fois plus de chances d'être séropositifs à Brucella que les travailleurs d'abattoirs (OR 1,93, IC à 95 % : 0,94-3,93, p = 0,07). Dans une analyse distincte des travailleurs de fermes ajustée uniquement en fonction de l'âge, le travail dans des fermes où les animaux sont en mauvaise santé, un test de lait positif, la participation à l'élevage de petits ruminants et l'aide à l'avortement du bétail étaient tous associés à des risques accrus de séropositivité. Les travailleurs qui consommaient du lait cru au moins une fois par mois étaient plus susceptibles d'être séropositifs (OR 3,79, IC à 95 % : 2,34-6,13, p < 0,001) tandis que les participants qui consommaient du lait fermenté et du fromage avaient plus de chances d'être séropositifs pour C. burnetii (OR 1.59 : IC à 95 % 1.26-2.00, p<0.001, et OR 1,70, IC à 95 % : 0,97-2,98, p=0,07 respectivement).

Conclusions :

Les éleveurs de bétail de la zone périurbaine de Lomé sont exposés aux bactéries Brucella spp. et Coxiella burnetii. La consommation généralisée de produits laitiers non bouillis et le manque d’utilisation d’équipements de protection individuelle (EPI) sont préoccupants, car il a été constaté que la consommation de produits laitiers et la participation à des activités d’élevage augmentent les risques de séropositivité pour les deux agents pathogènes. Une priorisation des maladies zoonotiques selon le principe ‘Une Santé’ permettrait de rassembler les secteurs concernés afin de ressourcer de manière adéquate la prévention et le contrôle des zoonoses prioritaires au Togo, qui peuvent avoir un impact particulier sur les travailleurs en contact étroit avec les animaux.

**Résumé de l'auteur :**

La santé humaine et la santé animale sont inextricablement liées, en particulier pour ceux qui vivent et travaillent en étroite collaboration avec les animaux. La brucellose et la fièvre Q sont deux maladies zoonotiques transmises par contact avec les animaux et par la consommation de produits laitiers, qui provoquent des fièvres non spécifiques et pour lesquelles les tests de diagnostic manquent dans de nombreux contextes de pays à revenu faible ou intermédiaire. Des études antérieures ont montré que les deux bactéries circulent dans le bétail au Togo. Nous avons mené une enquête auprès des travailleurs des fermes laitières et des abattoirs de la zone périurbaine de Lomé, au Togo, et avons constaté que 9,5 % et 28 % des travailleurs étaient respectivement séropositifs à Brucella et C. burnetii. Nous avons constaté que les facteurs de risque comprenaient les pratiques d’élevage et la consommation de produits laitiers. Des pratiques d’atténuation telles que l’utilisation d’EPI et l’ébullition du lait avant la consommation ou transformation sont des moyens simples par lesquels les éleveurs pourraient se protéger contre ces maladies zoonotiques et d’autres. Une priorisation systématique des zoonoses au Togo selon le principe ‘Une Santé’ permettrait une approche multisectorielle et une allocation adéquate des ressources pour la prévention et le contrôle de ces maladies.
